# RNA activation of haploinsufficient *Foxg1* gene in murine neocortex

**DOI:** 10.1038/srep39311

**Published:** 2016-12-20

**Authors:** Cristina Fimiani, Elisa Goina, Qin Su, Guangping Gao, Antonello Mallamaci

**Affiliations:** 1 Lab of Cerebral Cortex Development, SISSA, via Bonomea 265 - 34136 Trieste, Italy; 2 Viral Vector Core, University of Massachusetts Medical School - 368 Plantation Street, AS6-2049 - Worcester, MA 01605, USA; 3 Horae Gene Therapy Center, University of Massachusetts Medical School - 368 Plantation Street, AS6-2049 - Worcester, MA 01605, USA; 4 Department of Microbiology and Physiological Systems, University of Massachusetts Medical School - 368 Plantation Street, AS6-2049 - Worcester, MA 01605, USA

## Abstract

More than one hundred distinct gene hemizygosities are specifically linked to epilepsy, mental retardation, autism, schizophrenia and neuro-degeneration. Radical repair of these gene deficits via genome engineering is hardly feasible. The same applies to therapeutic stimulation of the spared allele by artificial transactivators. Small activating RNAs (saRNAs) offer an alternative, appealing approach. As a proof-of-principle, here we tested this approach on the Rett syndrome-linked, haploinsufficient, *Foxg1* brain patterning gene. We selected a set of artificial small activating RNAs (saRNAs) upregulating it in neocortical precursors and their derivatives. Expression of these effectors achieved a robust biological outcome. saRNA-driven activation (RNAa) was limited to neural cells which normally express *Foxg1* and did not hide endogenous gene tuning. saRNAs recognized target chromatin through a ncRNA stemming from it. Gene upregulation required Ago1 and was associated to RNApolII enrichment throughout the *Foxg1* locus. Finally, saRNA delivery to murine neonatal brain replicated *Foxg1*-RNAa *in vivo*.

More than one hundred different hemizygous gene deletions underlie a variety of neuropathological conditions, leading to epilepsy, mental retardation, autism, schizophrenia and neurodegeneration^1^,^2^,^3^,^4^,^5^. Their individual prevalence is low, however their cumulative frequency makes them an issue for social health. A scalable therapeutic approach is needed.

How to achieve this goal? In principle, homologous recombination (HR)-mediated repair of defective genes, triggered by Engineered EndoNucleases (EEN) and driven by a dedicated editor DNA, should be the golden procedure to fix the problem^6^,^7^,^8^. In reality, the implementation of this approach within the CNS would be hardly feasible, due to a variety of technical issues^9^,^10^,^11^. A more spartan design, based on therapeutic minigenes, would be problematic as well. In fact, the exact rescue of gene expression levels is often needed for proper execution of neural gene functions^12^,^13^,^14^. Moreover, the faithful recapitulation of the gene expression pattern generally requires a number of properly arranged cis-active elements. Clustering all of them into a small transgene, suitable for panneural delivery, can be hardly feasible and/or scalable. Therefore, a different approach is needed. This might be a gentle stimulation of the spared gene allele, still under the control of the regulatory elements which shape its baseline expression profile and mediate subtle modulation of its levels linked to neuronal physiology.

Nowadays, two classes of molecular tools are potentially available for this last approach: (a) artificial transcription factors, and (b) small activating RNAs (saRNAs). The former ones include Zinc finger- (ZF-)^15^,^16^,^17^,^18^,^19^, TransActivator Like Element- (TALE-)^20^,^21^,^22^, Clustered Regularly Interspaced Short Palindromic Repeat (CRISPR)-23,24,25,26,27,28 and NMHV-type^29^ transactivators. Despite their capability to stimulate endogenous genes ad libitum, their employment for therapy of neural haploinsufficiencies may be problematic, because of their very large size^30^ and ectopic gene activation^26^,^27^,^28^,^31^. saRNAs, i.e. miRNA/siRNA-like molecules targeting the surroundings of the transcription unit in order, may also stimulate transcription, upon delivery as mature moieties, pri-miRNA- or shRNA precursors. As such, they work as effectors of “RNA(-dependent gene) activation” (RNAa)^32^,^33^,^34^. After the initial RNAa report by Li *et al*.^35^, several genes were transactivated by saRNAs^32^,^33^,^34^. Despite the intricate and heterogenous epigenetic changes evoked by these effectors^36^, their ultimate functional outcome seems to be basically attributable to two distinct molecular mechanisms. saRNAs can act by destabilizing ncRNAs which normally dampen mRNA transcritpion. Alternatively, they can convey the transcriptional machinery to chromatin^37^,^38^. Interestingly, moderate power and small size of saRNAs make them a promising tool for treatment of neural haploinsufficiencies. However their biological effectiveness, selectivity and compliance with endogenous gene tuning still wait for in depth characterization.

As a proof-of-principle, here we employed RNAa to stimulate the haploinsufficient *Foxg1* transcription factor gene. *Foxg1* is a key regulator of cortico-cerebral development and function, implicated in pallial field specification^39^, precursors proliferation control^40^,^41^ and laminar^42^ as well as areal^43^ neuronal differentiation. In humans, its allele dosage is crucial to neurological health, as hemideletion and duplication of *Foxg1* lead to Rett and West syndromes, respectively^44^. Briefly, we found that RNAa resulted into a *Foxg1* expression gain suitable for therapeutic purposes and led to an appreciable biological outcome. No ectopic gene activation occurred and endogenous gene tuning was preserved. Finally, a robust *Foxg1* stimulation was also achieved *in vivo*.

## Results

### Selecting miRNA-like saRNAs upregulating *Foxg1*-mRNA

**Figure 1:**
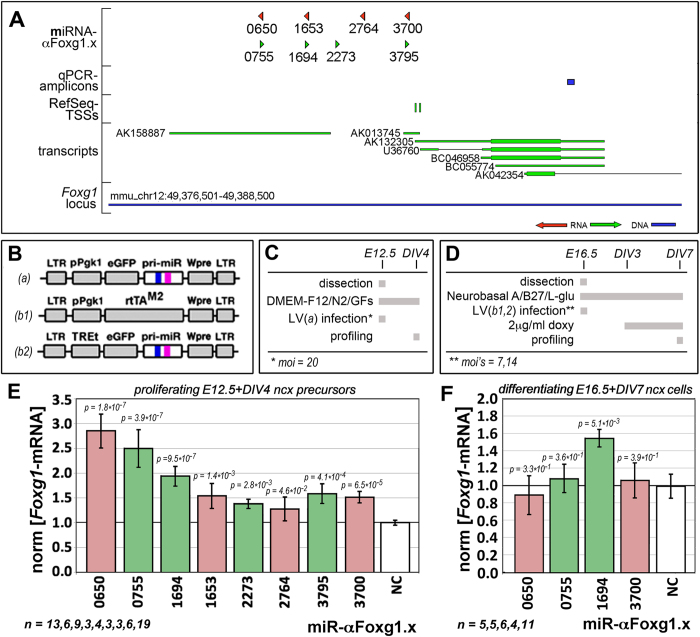
Screening for miRNA-like, small RNAs activating *Foxg1*-mRNA (*Foxg1*-saRNAs) in murine neocortical precursors and derivatives. (**A**) Schematics of the *Foxg1* locus including saRNA positions and orientations as well as the diagnostic qRTPCR amplicon. (**B–D**) Lentiviral reagents and protocols employed for this screening. (**E,F**) *Foxg1*-mRNA levels in proliferating neocortical precursors and their differentiating derivatives, manipulated as in (**C**) and (**D**), respectively. Values double normalized, against *Gapdh* and control (NC). E, embryonic day. DIV, days *in vitro*. Bars represent sem’s. *n* = number of biological replicates. *p*-values were calculated by the t-Student algoritm (one-tail, unpaired). All results with *p* < 0.05 further passed Benjamini-Hochberg filtering, with *FDR* < 1/m.

In order to identify potential genomic targets appropriate for *Foxg1*-RNAa, we inspected the 5′ surroundings of NCBI-RefSeq *Foxg1*-mRNA transcriptional start sites (TSSs) for sequences specifically amenable to miRNA targeting, via the pri-miRNA-155-based Block-It platform^45^. We selected eight high-score candidates (Fig. 1A and Supplementary Table 1) and we cloned the cDNAs, encoding for the corresponding precursors, into the lentiviral constitutive expressor pLVmiR.23^45^ (Fig. 1B). We acutely delivered the resulting lentiviruses to murine E12.5 neocortical precursors, we kept these cells as floating neurospheres in pro-proliferative medium for four days and we eventually scored them for *Foxg1*-mRNA levels by qRTPCR (Fig. 1C). We found that 8 out of 8 miRNAs, 4 antisense-oriented (miR-αFoxg1.0650, miR-αFoxg1.1653, miR-αFoxg1.2764 and miR-αFoxg1.3700) and 4 sense-oriented (miR-αFoxg1.0755, miR-αFoxg1.1694, miR-αFoxg1.2273 and miR-αFoxg1.3795), upregulated *Foxg1*, to different extents. The expression gain ranged from 1.28 ± 0.24-folds (miR-αFoxg1.2764) to 2.88 ± 0.34-folds (miR-αFoxg1.0650) (Fig. 1E and Supplementary Table 2). Similar results were achieved upon delivery of miR-αFoxg1.0650 and miR-αFoxg1.1694 to NIH3T3 and HEK293T cells, which led to a consistent increase of Foxg1/FOXG1 proteins (Fig. S1 and Supplementary Table 2).

Next, we wondered if *Foxg1*-RNAa may be also achieved in differentiating derivatives of neocortical precursors. We transferred the pri-miRNA-cDNAs of the four best-performing miRNAs (Fig. 1E) into LV_TREt-IRES2EGFP^46^, inbetween the doxycyclin-controlled TREt promoter and an IRES2EGFP reporter gene (Fig. 1B,(b2)). We employed the resulting lentiviruses - paired to a constitutive rtTA^2S^-M2 transactivator expressor, LV_Pgk1p-rtTA^2S^-M2^46^ (Fig. 1B,(b1)), to drive delayed, TetON-controlled miRNA expression. Unexpectedly, we found that only one miRNA (miR-αFoxg1.1694) upregulated *Foxg1*, by 1.56 ± 0.11-folds. The other ones were uneffective (Fig. 1F and Supplementary Table 2).

**Figure 2:**
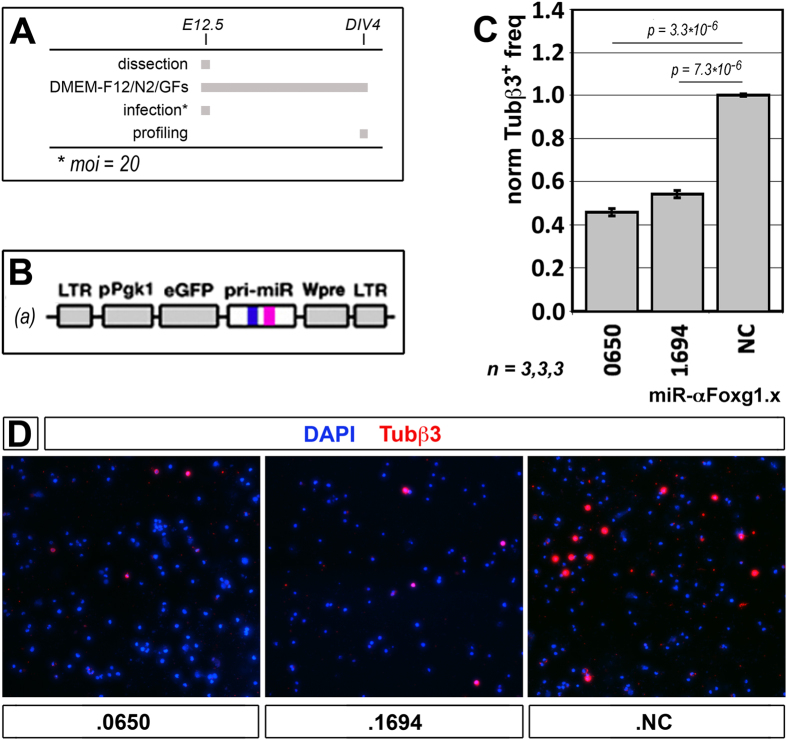
Histogenetic outcome of *Foxg1*-RNAa. (**A,B**) Protocols and lentiviral reagents employed for this assay. (**C**) Quantification of cells immunopositive for the neuron-specific Tubβ3 marker, in cultures of neocortical precursors expressing *Foxg1*-saRNAs. (**D**) Examples of Tubβ3^+^ immuno-fluorescences referred to in (**C**). E, embryonic day. DIV, days *in vitro*. Bars represent sem’s. *n* = number of biological replicates. Statistical significance of results evaluated by t-Student assay (one-tail, unpaired). Absolute average frequency of Tubβ3^+^ cells in NC samples was (27.25 ± 0.16)%.

Finally, to corroborate the significance of these results, we tested if the small expression gain elicited by our saRNAs led to an appreciable biological readout. For this purpose, we stimulated *Foxg1* by miR-αFoxg1.0650 and .1694 in proliferating murine neocortical precursors (Fig. 2A,B) and we evaluated the impact of this manipulation on the generation of postmitotic, Tubβ3^+^ neurons. *Foxg1* - in fact - inhibits the exit of neuronogenic precursors from cell cycle^40^,^41^ and even a small increase of its expression level is known to exert a deep impact on neuronogenic differentiation rates^29^. As expected, both miRNAs halved the neuronal output of the culture, in a highly reproducible fashion (Fig. 2C,D and Supplementary Table 2).

### Compliance of RNAa with endogenous tuning of *Foxg1*-mRNA

**Figure 3:**
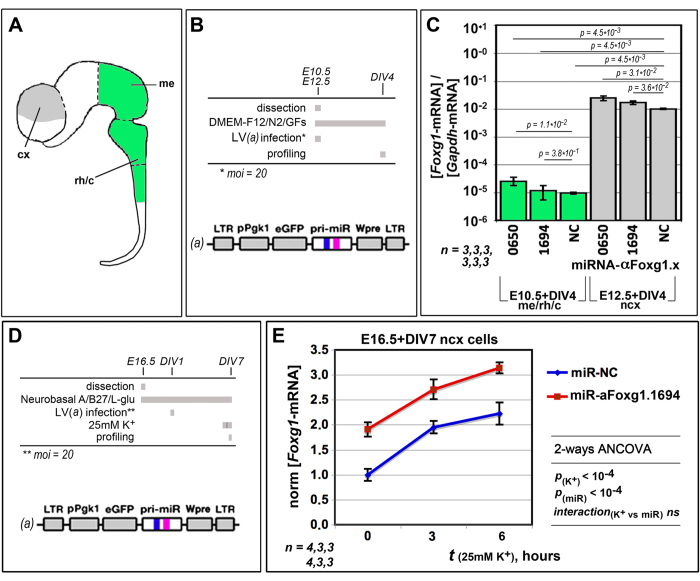
Compliance of *Foxg1*-RNAa with endogenous gene regulation. (**A**) Idealized representation of the murine early neural tube, including cortical (cx), mesencephalic (me) and rhombo-cervical (rh/c) domains. (**B**) Protocols and lentiviral reagent employed for the assay referred to in (**C**). (**C**) Impact of miR-αFoxg1.0650 and 0.1694 on *Foxg1*-mRNA levels in proliferating precursors from the me/rh/c and cx domains. (**D**) Protocols and lentiviral reagent employed for the assay referred to in (**E**). (**E**) *Foxg1*-mRNA modulation by miR-αFoxg1.1694 in differentiating neocortical derivatives upon their timed terminal exposure to 25 mM K^+^. E, embryonic day. DIV, days *in vitro*. Bars represent sem’s. *n* = number of biological replicates. Statistical significance of results evaluated by t-Student (one-tail, unpaired) (**C**) and ANCOVA (two-ways, unpaired) (**E**) assays. *ns*, not significant.

The therapeutic exploitation of RNAa for the treatment of haplo-insufficiencies would be easier if the *activity* of saRNAs would be confined to cells normally expressing the gene in order. To assess the fulfilment of this requirement, we delivered miR-αFoxg1.0650 and .1694 to proliferating neural precursors originating from the murine E10.5 meso-rhombo-cervical neural domain, which does not express *Foxg1*^47^. We employed neural precursors dissected out of the E12.5 neocortex as histogenetically-equivalent positive controls (Fig. 3A). Interestingly, albeit weakly upregulated by miR-αFoxg1.0650 and .1694, *Foxg1* levels remained about 3 orders of magnitude lower in meso-rhombo-cervical derivatives, compared to neocortical controls (Fig. 3B,C and Supplementary Table 2). This suggests that risks of ectopic gene activation upon RNAa can be negligible.

Neuronal genes often undergo fine, electrical activity-related tuning, which may be crucial to proper implementation of their function^48^. An acceptable therapy of neuropathogenic haploinsufficiencies relying on stimulation of the spared gene allele should take into account such physiological gene modulation. Exposure of neocortical neurons to high extracellular [K^+^] was followed by a prompt arousal of *Foxg1*-mRNA levels (Fig. 3D,E and Supplementary Table 2), a likely *in vitro* correlate of activity-dependent *Foxg1* stimulation. We reasoned that this phenomenon might provide a valuable opportunity for probing compliance of RNAa with “endogenous” gene tuning. Remarkably, the delivery of miR-αFoxg1.1694 to K^+^-challenged neocortical neurons elicited a delicate upward shift of the *Foxg1* activation curve under high extracellular [K^+^]. However, ANCOVA analysis of data provided no evidences of interaction between K^+^ stimulation and RNAa (Fig. 3E), suggesting that RNAa does not hide activity-driven *Foxg1* tuning.

### Molecular mechanisms underlying *Foxg1*-RNAa

**Figure 4:**
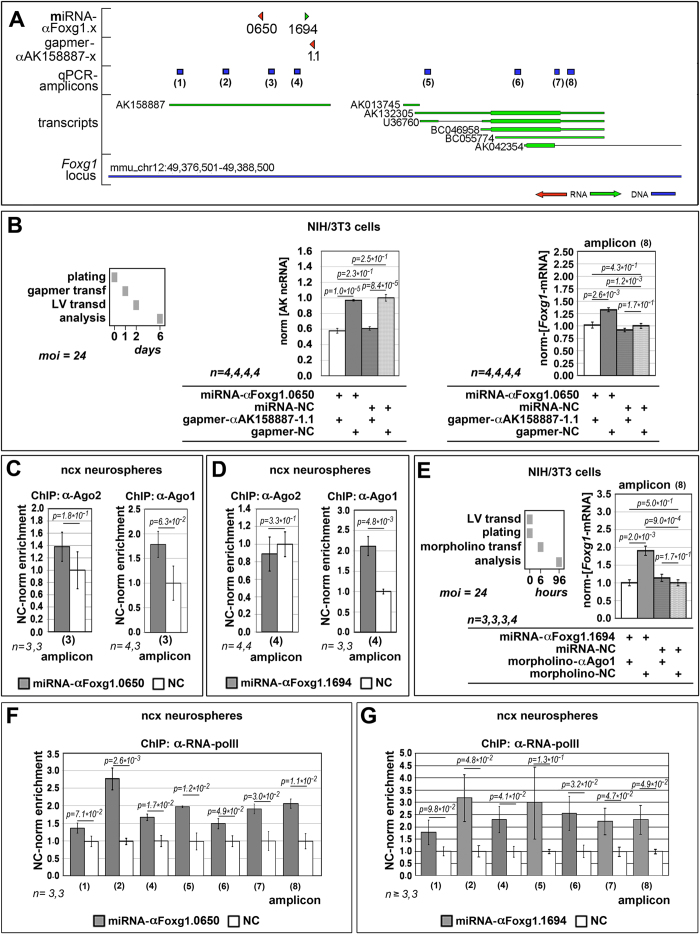
Molecular mechanisms underlying *Foxg1*-RNAa. (**A**) Schematics of the *Foxg1* locus including miRNA and gapmer positions and orientations, as well as diagnostic qPCR amplicons. (**B**) AK158887-ncRNA and *Foxg1*-mRNA levels in NIH/3T3 cells upon combined delivery of miR-αFoxg1.0650 and gapmer-αAK158887-1.1. Values double normalized, against *Gapdh* and control (NC). (**C,D**) qPCR quantification of *Foxg1* chromatin enrichment, upon immunoprecipitation (ChIP) by antibodies against Argonaute 2 (α-Ago2) and Argonaute 1 (α-Ago1). Evaluation performed in neocortical precursors challenged by miR-αFoxg1.0650 (**C)** and miR-aFoxg1.1694 (**D**), according to the protocol shown in Fig. 1B,C. Values double normalized against input chromatin and control (NC). (**E**) *Foxg1*-mRNA levels in NIH/3T3 cells upon combined delivery of miR-αFoxg1.1694 and morpholino-αAgo1. Values double normalized, against *Gapdh* and control (NC). (**F**,**G**) qPCR quantification of *Foxg1* chromatin enrichment, upon ChIP by antibodies against RNA polymerase II (α-RNA-polII). Evaluation performed in neocortical precursors challenged by miR-αFoxg1.0650 (**F**) and miR-aFoxg1.1694 (**G**), according to the protocol shown in Fig. 1B,C. Values double normalized against input chromatin and control (NC). Bars represent sem’s. *n* = number of biological replicates. *p*-values were calculated by the t-Student algoritm (one-tail, unpaired). All panel 4 F results with *p* < 0.05 further passed Benjamini-Hochberg filtering, with *FDR* < 1 /m. The same applies to panel 4 G, except amplicon^6^ results.

RNAa is supposed to be a heterogeneous process and at least two classes of molecular mechanisms are supposed to underlie it. RNAa may take place via downregulation of ncRNAs which limit transcription of the associated gene of interest. Alternatively, saRNAs may drive molecular machinery promoting transcription to target chromatin^37^,^38^. To cast light on this issue, we monitored expression levels of the *Foxg1*-associated, sense-oriented AK158887 ncRNA (Fig. 4A), following the delivery of antisense-oriented, miR-αFoxg1.0650 and miR-αFoxg1.1653. No down-regulation of AK158887 was found, suggesting that, at least in these cases, the latter mechanism may apply (Fig. S2).

As for recognition of target chromatin, saRNAs might straightly bind to unwound chromosomal DNA. Alternatively, they might pair to nascent RNA molecules stemming from it. To distinguish between these possibilities, we downregulated the putative miR-αFoxg1.0650 target AK158887 RNA, by gapmer-αAK158887-1.1 in easily transfectable NIH/3T3 cells Fig. 4A,B). Interestingly, such manipulation fully abolished miR-αFoxg1.0650-dependent *Foxg1* transactivation (Fig. 4B), while not affecting *Foxg1* levels in miRNA-NC-treated samples. This suggests that miR-αFoxg1.0650 recognizes its chromatin target via RNA/RNA pairing.

Both Ago1 and Ago2 are detectable in the nucleus and can bind miRNAs^49^. Ago2 was also specifically implicated in a number of RNAa cases, possibly acting as a bridge between the saRNA and the supramolecular transactivating complex^50^. To assess the involvement of Ago2 in *Foxg1*-RNAa, we evaluated its recruitment to miR-αFoxg1.0650 and .1694 target sequences, upon saRNA delivery to neocortical precursors, by ChIP. Enrichment for Ago1 was monitored as a specificity control. Unexpectedly, both saRNAs increased the recruitment of Ago1, but not of Ago2 (Fig. 4C,D), pointing to a selective involvement of the former in *Foxg1*-RNAa. To corroborate this inference, we antagonized Ago1 translation by a dedicated morpholino in NIH/3T3 cells (Fig. 4E). Remarkably, this treatment suppressed miR-αFoxg1.1694-dependent *Foxg1* transactivation (Fig. 4E), while not affecting *Foxg1* levels in miRNA-NC-treated samples. All this confirms the pivotal role of Ago1 in *Foxg1*-RNAa.

To further explore mechanisms leading to RNAa, we monitored the enrichment of the *Foxg1* locus for RNApolII, upon saRNA delivery to neural precursors. We found that both miR-αFoxg1.0650 and 0.1694 robustly increased RNApolII recruitment along the entire locus (Figs 4A,F,G and Supplementary Table 2), which likely led to augmented transcription rates. Intriguingly, the absolute RNApolII recruitment profile did not display any sudden decrease downstrem of *Foxg1*-TSS in control conditions (Fig. 4A and S3, and Supplementary Table 2). Moreover, no abrupt increase of RNApolII recruitment took place in the same position upon saRNA delivery (Fig. 4A,F,G, and Supplementary Table 2). Altogether these data suggest that RNApolII does not normally pause near *Foxg1*-TSS and saRNAs stimulate *Foxg1* transcription by promoting RNApolII recruitment to TSS.

### *In vivo* Foxg1-RNAa

Although highly flexible and powerful for analytical purposes, lentiviral vectors employed throughout this study would pose obvious concerns for *in vivo* exploitation, due to their insertional mutagenesis activity. In principle, we could circumvent this issue replacing lentivirus-encoded saRNAs by their synthetic siRNA-like analogues. To preliminarily explore this possibility, we delivered siRNA-αFoxg1.1694 (a synthetic analogue of miR-αFoxg1.1694) or the siRNA-αGFP control to murine neocortical differentiating derivatives, by Lipofectamine-RNAimax transfectant. Interestingly, siRNA-αFoxg1.1694 specifically upregulated *Foxg1*-mRNA, by 1.47 ± 0.02-folds (Fig. S4A,B and Supplementary Table 2). Encouraged by this result, we repeated this *in vitro* assay, replacing the commercial transfectant by the Chimeric Rabies Virus Glycoprotein Fragment (RVG-R9). This polypeptide may be easily loaded with nucleic acids, it crosses the blood-brain-barrier (BBB) and targets the vast majority of CNS cells via a specific interaction with the α7 chain of the nicotinic receptor^51^. As such, it is a promising tool for therapeutic brain targeting. Interestingly, RVG-R9-mediated siRNA-αFoxg1.1694 transfection replicated *Foxg1*-mRNA upregulation obtained by RNAimax (Fig. S4C,D and Supplementary Table 2). Given the documented expression of α7 in proliferating neocortical precursors^52^, we tested if the RVG-R9/siRNA-αFoxg1.1694 complex might upregulate *Foxg1* even in neurospheres originating from E12.5 cortical tissue. Unfortunately, the huge upregulation detected (almost 9-folds) was not specific, since it was also achieved by the RVG-R9/siRNA-αGFP control complex (Fig. S4E,F and Supplementary Table 2). Even worse, such upregulation was associated to massive differentiation of proliferating precursors to postmitotic neurons (Fig. S4G). Therefore, *in vivo* employment of RVG-R9 might lead to a detrimental precocious exhaustion of neuronogenic niches. Because of that, we considered an alternative delivery tool for our saRNAs.

**Figure 5:**
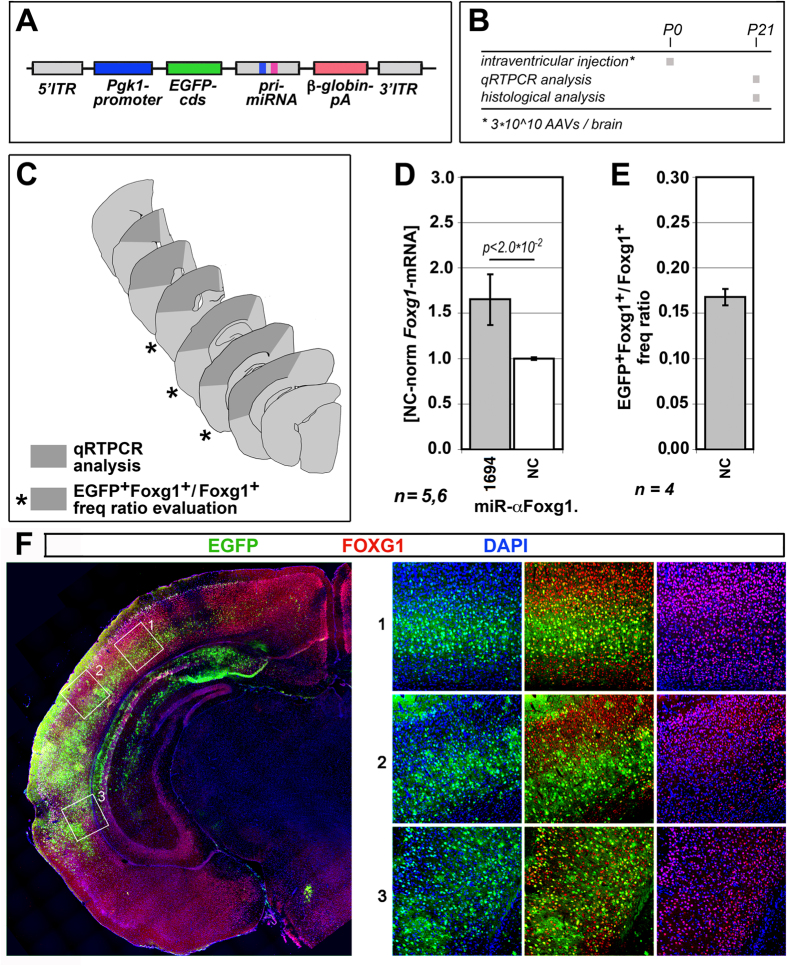
*Foxg1*-RNAa in murine neocortex. **(A)** Schematics of AAV9-pseudotyped, self-complementary AAV2-derivative, adeno-associated viral vector, driving constitutive expression of *Foxg1*-activating miRNAs. (**B**) Protocol employed for the assays referred to in (**C**–**F**). (**C**) Location of neocortical sectors subject of the analyses shown in (**D**–**F**). (**D**) Quantification of *Foxg1*-mRNA levels in neocortex of juvenile mice previously injected by scAAVs encoding for miR-αFoxg1.1694. (**E**) Evaluation of frequency of Foxg1^+^ cells transduced by EGFP-encoding control virus (NC). (**F**) Examples of αFoxg1/αEGFP-immunoprofiled sections referred to in (**C**,**E**). P, post-natal day. Bars represent sem’s. *n* = number of biological replicates (i.e. brains). Statistical significance of results evaluated by t-Student assay (one-tail, unpaired).

We chose to administer miR-αFoxg1.1694 to the living brain through AAV9-pseudotyped, self-complementary AAV2-derivative, adeno-associated viral vectors, under the control of a constitutive promoter (Fig. 5A). We injected 3*10^10^ infecting particles into the right lateral ventricle of P0 mouse pups by free hands. We sacrificed these animals three weeks later (P21) and scored their right neocortices for *Foxg1*-mRNA content as well as for the frequency at which Foxg1^+^ cells were AAV-transduced (Fig. 5B,C). Remarkably, *Foxg1* was upregulated by 1.66 ± 0.30 folds (Fig. 5D and Supplementary Table 2), even though the transduction frequency of Foxg1^+^ cells was only 0.17 ± 0.01 (Fig. 5E,F).

## Discussion

Active in telencephalic precursors and their postmitotic derivatives, the brain patterning gene *Foxg1* controls a variety of neurodevelopmental and physiological processes. Its allele dosage is crucial, since its duplication and deletion result in West and Rett-like syndromes, respectively. As a proof-of-principle of RNAa therapy of *Foxg1* haploinsufficiency, here we selected 8 artificial saRNAs upregulating *Foxg1* in neocortical precursors, 1 of which worked in their postmitotic derivatives too (Fig. 1). Expression of these saRNAs elicited an appreciable biological outcome (Fig. 2). RNAa was restricted to neural cells expressing the target gene and did not interfere with its endogenous tuning (Fig. 3). saRNAs recognized their target chromatin through nascent ncRNAs and recruited RNApolII to it, possibly via Ago1 (Fig. 4). Finally, delivery of one saRNA to mouse neonates, by intraventricular injection of recombinant AAV vectors, replicated *Foxg1*-RNAa *in vivo* (Fig. 5).

Interestingly, only one out of the best four miRNAs activating *Foxg1* in proliferating neocortical precursors worked satisfactorily in their postmitotic derivatives (Fig. 1A,E,F). This may be due to the different epigenetic state of chromatin, generally more accessible in the former ones^53^,^54^. It may specifically reflect a different ncRNA landscape at the *Foxg1* locus. Despite the moderate amplitude of *Foxg1*-upregulation achieved by RNAa, such manipulation elicited a pronounced histogenetic effect (Fig. 2). This was not a surprise. A high sensitivity of neuronogenic rates to even subtle changes of *Foxg1* levels was already reported^29^. Moreover, similar phenomena were described for a number of other patterning genes, including *Emx2* and *Pax6*^45^,^55^,^56^.

We also found that the saRNAs achieved a relevant molecular outcome only in primary cultures where the gene of interest was active (Fig. 3A–C). This suggests that therapeutic saRNA delivery, driven by a ubiquitous promoter or achieved via straight administration of pre-made, synthetic molecules, should be followed by the activation of the target gene limited to its standard expression domain. Moreover, within responsive neurons, saRNAs elicited a gentle and reproducible stimulation of the gene in order, which did not interfere with its fine endogenous tuning (Fig. 3D,E). All that strenghtens the saRNA suitability for precise and affordable treatment of haploinsufficiences, with special emphasis on those of neurological interest.

Concerning mechanisms of RNAa, the employment of gapmers against ncRNAs stemming from the target locus is an elegant method for unveiling its molecular logic. Specifically, if the gapmer reproduces the saRNA effect, then gene activation should originate from destabilization of its ncRNA target, as described for *Bdnf* by ref. 57. If the gapmer suppresses saRNA activity - as reported for *PR* and *COX2* by refs 38 and 50 - then RNAa should rather rely on the recruitment of transactivating effectors to the target locus, via ncRNA docks stemming from it. The latter scenario is what we observed for *Foxg1* upon delivery of miR-αFoxg1.0650 (Fig. 4B). Other antisense saRNAs stimulating this gene might work in a similar way. Sense-oriented saRNAs might land on not yet mapped, *Foxg1*-associated antisense-ncRNAs, or act according to a different molecular logic.

Beyond target chromatin recognition by saRNAs, a crucial role in RNAa is played by Argonautes, which act as adaptors between the chromatin-bound saRNAs and the effector complex stimulating transcription. In a number of cases, Ago2 was reported to be the key player. It binds the target gene through saRNAs, it mediates the assembly of a supramolecular dock for RNApolII, and it is ultimately necessary for RNAa^35^,^50^,^58^,^59^,^60^. Ago1 binds to TSS surroundings too. Moreover, it interacts with RNApolII and is involved in transcription regulation^49^. However, initial reports implicated it in transcriptional gene silencing (TGS) rather than RNAa^35^,^58^. Unexpectedly, we found that suppressing Ago1 by morpholino abolished *Foxg1*-RNAa (Fig. 4E). This phenomenon is consistent with the recruitment of Ago1 to the *Foxg1* promoter, triggered by saRNAs (Fig. 4D). It echoes the recent report of Ago1-dependent RNAa at the *IL2* locus^61^.

A step further along the RNAa cascade, RNApolII is recruited to TSS^49^,^58^,^59^,^62^,^63^,^64^ or possibly stimulated to progress downstream of it^65^. In case of *Foxg1-*mRNA, the RNApolII enrichement profile of the gene, in baseline conditions as well as upon miR-αFoxg1.0650 and .1694 delivery (Figs S3 and 4F,G), suggests that the former mechanism applies.

miR-αFoxg1.1694 worked also *in vivo* (Fig. 5). Here, the cumulative *Foxg1* expression gain was about +68%, albeit only 1/6 of Foxg1-expressing cells were targeted. This means that the actual expression gain in targeted Foxg1^+^ cells might be not far from 6*68%, i.e. about +400%. This suggests that, in a therapeutic scenario, saRNA expression should be dampened to restore physiological *Foxg1*-mRNA expression levels, possibly via a weaker promoter or a tunable transactivating system. Moreover, the employment of more advanced AAV drivers^66^ might help targeting the almost totality of telencephalic neural cells.

In summary, we have selected a set of artificial miRNA eliciting a gentle *Foxg1* transactivation, specifically in cortico-cerebral cells. Their delivery led to an appreciable biological outcome, while complying with endogenous gene tuning. They stimulated RNApolII recruitment, possibly via Ago1. One of these miRNAs worked promisingly *in vivo*, even though its therapeutic employment still requires further optimization. As recently shown, hemizygosity for specific genes and polygenic chromosomal segments underlies a huge number of neuropathological entitites^1^,^2^,^3^,^4^,^5^, for which no cure are presently available. Based on results reported above, RNAa might be a simple and scalable approach for fixing this class of problems.

## Materials and Methods

### Animal handling

Wild-type, CD1 strain mice used in this study were purchased from Envigo-Italy and housed at the SISSA mouse facility. Animals handling and subsequent procedures were in accordance with European [European Communities Council Directive of November 24, 1986 (86*/*609*/*EEC)] and Italian laws (D.L. 04.03.2014, n°26) and were approved by SISSA Board for Animal Welfare. Embryos were staged by timed breeding and vaginal plug inspection. Neonates were staged as “P0” on their birthday.

### Cell cultures

#### Embryo harvesting

Embryos (E10.5, E12.5 and E16.5) were harvested from pregnant dams killed by cervical dislocation and put in sterile ice-cold PBS supplemented with 0.6% glucose. Cerebral cortices (E12.5 and E16.5), mesencephalons (E10.5) and rhombocervical tracts (E10.5) were then dissected and collected in the same solution.

#### Primary cells

E12.5 cerebral cortices as well as E10.5 mesencephalons and rhombo-cervical tracts were mechanically dissociated to single cells by gentle pipetting. Neural precursor cells were subsequently counted in a Burker chamber and plated in 24-multiwell plates (Falcon), at the density of 1,000 cells*/*μl, in proliferative medium [DMEM-F12 (Gibco), 1X Glutamax (Gibco), 1X N2 (Invitrogen), 1 mg*/*ml BSA, 0.6% glucose, 2 μg*/*ml heparin (Stem Cell Technologies), 20 ng*/*ml bFGF (Invitrogen), 20 ng*/*ml EGF (Invitrogen), 1X Pen- Strept (Gibco), 10 pg*/*ml Fungizone (Gibco)]. Neural precursors were acutely infected by recombinant lentiviruses and kept in culture up to 96 h. Multiplicities of infection (moi’s) are reported in the corresponding figures.

Cortical tissue from E16.5 mice was chopped to small pieces for 5 minutes, in the smallest volume of ice-cold 1X PBS-0,6% glucose-1 mg/ml DNaseI. The minced tissue was then resuspended and digested in 0.25 mg/ml trypsin-1mg/ml DNAseI for 5 minutes at 37 °C. Digestion was stopped by adding ≥1.5 volumes of DMEM/F12/10%FBS. Cortical tissue was spinned down and transferred to differentiative medium. The suspension was pipetted 5–8 times with a P1000 Gilson pipette and undissociated tissue was left to sediment for 1–2 minutes. The supernatant was harvested and the living cells counted. 1 × 10^6 cells/well were plated on poly-L-Lysine coated 12 multiwell plates, in 600 μl of differentiative medium [Neurobasal-A (Gibco), 1X Glutamax (Gibco), 1X B27 supplement (Invitrogen), 25 μM L-glutamate, 25 μM β-Mercaptoethanol (Gibco), 2% FBS, 1X Pen/Strept (Gibco), 10 pg/ml Fungizone (Gibco)]. Dissociated neural cells were infected 24 hours later and kept in culture up to 7 days. Multiplicities of infection (moi’s) are reported in the corresponding figures. When required, doxycycline was added to the culture medium, at 2 μg/ml. Medium was half-replaced with fresh one every 3.5 days.

#### HEK293T and NIH/3T3 cells

Cells were cultured in DMEM-Glutamax^TM^ (Gibco)-10% FBS, at 125,000 and 25,000 cells/cm^2^, respectively, according to standard protocols. Lentiviral transductions were performed at moi’s reported in the corresponding figures. When appropriate, αAgo1 and αGFP morpholinos (GeneTools) were delivered to NIH/3T3 cells at 10 μM, by 6 μM EndoPorter^TM^ carrier (GeneTools), according to manufacturer’s instructions. When appropriate, αAK158887-1.1 or control Antisense LNA GapmeRs (Exiqon) were delivered to cells at 50 nM, by Lipofectamine 3000 reagent (TermoFisher), according to manufacturer’s instructions.

### Selection of candidate saRNAs

cDNAs encoding for pri-miRNAs targeting the *Foxg1* locus were designed using “BLOCK-iT™ RNAi Designer” (Invitrogen). This is a proprietary, freely online accessible program, conceived for selection of pri-miRNA-155-based, artificial miRNAs to be employed for gene knock-down. We repurposed it for designing potential small miRNA-like activators of *Foxg1* expression. The 4kb genomic region extending from −3.8 kb to +0.2 kb with respect to the 5′ *Foxg1*-mRNA TSS (Fig. 1A) was scanned in 0.5kb frames, in both sense and antisense orientation. Candidate miRNAs with a score ≥4.5/5 were shortlisted. They were further filtered for absence of potential off-targets within the murine genome and transcriptome, by Blat (UCSC) and Blastn (NCBI) softwares, respectively. A subset of them, recognizing targets evenly distributed within the 4.0 kb reference region and including hits with different homologies to their human counterparts, was selected. A summary of these candidate miRNAs and their key parameters is provided in Supplementary Table 1.

### Lentiviral vector construction

cDNAs encoding for pri-miRNAs targeting the *Foxg1* locus were designed using “BLOCK-iT™ RNAi Designer” (Invitrogen). Genomic locations of their targets are listed in Supplementary Table 1. The negative control pri-miRNA-cDNA derived from “pcDNA^TM^-6.2-GW/EmGFP-miR_neg_control_plasmid” (Invitrogen), as described in ref. 45. These pri-miRNA-cDNAs were cloned into BfuAI-digested pLVmiR.23^45^, so obtaining “LTR-pPgk1-eGFP-pri-miR-Wpre-LTR” constitutive expressors [Fig. 1(a)]. The TetON-controlled “LTR-TREt-eGFP-pri-miR-Wpre-LTR” pri-miRNA expressors [Fig. 1B (b2)] were obtained by transferring the AgeI/KpnI inserts originating from the corresponding constitutive expressors into AgeI/KpnI cut LV:TREt-IRES2-EGFP^67^. Finally, “LTR-pPgk1-rtTA^M2^-Wpre-LTR” [Fig. 1B(b1)] was described in ref. 68. For each construct, inserts and their surroundings were checked by double strand sequencing.

### Recombinant lentivirus production

Recombinant third generation self-inactivating (SIN) lentiviruses were produced and titrated as previously described^68^.

### RNA profiling

Total RNA was extracted from cells using TRIzol Reagent (Invitrogen) according to manufacturer’s instructions. Agarose gel electrophoresis and spectrophotometric measurements (NanoDrop ND-1000) were employed to estimate its concentration, quality and purity. RNA preparations were treated by TurboDNAseI kit (Gibco) 1 h at 37 °C. At least 0.5 μg of total RNA from each sample was retrotranscribed by SuperScriptIII^TM^ (Invitrogen) in the presence of random hexamers, according to the manufacturer’s instructions. 1*/*100 of the resulting cDNA was used as substrate of any subsequent qPCR reaction. Next, negative control PCRs were run on RT− cDNA preparations. In general, PCR reactions were performed by the SsoAdvanced SYBR Green Supermix^TM^ platform (Biorad), according to manufacturer’s instructions. For each transcript under examination and each sample, cDNA was PCR-analyzed in technical triplicate, against absolute standards, and average results calculated. Averages were normalized against *Gapdh* and further normalized against controls. Experiments were performed at least in biological triplicate and analyzed by Student’s t-test.

### Western Blotting

Western analysis was performed according to standard methods. Total cell lysates in CHAPS buffer were quantified by BCA protein assay kit (Fisher Scientific #10678484) and denatured at 95 °C for 5 min, prior to loading. Twenty-five micrograms of proteins were loaded per each lane of a 12% acrylamide−0.1% SDS gel. FOXG1/Foxg1 was detected by a primary rabbit anti- Foxg1 polyclonal antibody^41^, used at 1:2000, and a secondary HRP-conjugated anti-rabbit antibody (LifeTech #32260), used at 1:2000. βACT was detected by a peroxydase C-conjugated mouse monoclonal antibody (Sigma #A3854), used at 1:10 000. FOXG1/Foxg1 and βACT were sequentially revealed by an ECL kit (GE Healthcare # GERPN2109). Images were acquired by an Alliance LD2–77.WL apparatus (Uvitec, Cambridge) and analyzed by Adobe Photoshop CS2 software^TM^ and Microsoft Excel 11 software^TM^.

### ChIP-qPCR

The chromatin immunoprecipitation quantitative polymerase chain reaction assays (ChIP-qPCRs) were performed on chromatin extracted from neural cell cultures. Cells were acutely infected with bio-active and control lentiviruses. Then, they were kept in culture for 96 h. ChIP analysis was performed according to the MAGnify^TM^ Chromatin Immunoprecipitation System protocol (Invitrogen), with minor modifications. For each ChIP assay, chromatin from 10^6^ cells was fixed by 1% formaldehyde, for 10 min at RT. After cell lysis, fixed chromatin was sonicated by a Soniprep 150 apparatus into ~600 bp fragments (on ice; 5 s ON, 55 s OFF; oscillation amplitude 5 μm; 4 cycles). Sonicated chromatin was immunoprecipitated for 2 h at 4 °C, by 2.5 μg of an anti-RNApolII antibody (mouse clone 4H8, Abcam #ab5408), 2.5 μg of an anti-Ago1 (mouse clone 6D8.2, Millipore #04–083), 3.0 μg of an anti-Ago2 (rabbit polyclonal, Abcam #32381) or 2.5 μg of murine IgG (from MAGnify kit, Invitrogen), in a final volume of 100 μl. Immunoprecipitated DNA was purified according to the manufacturer’s instructions. Lastly, 1*/*60 of each immunoprecipitated (IP) DNA sample was amplified by qPCR. For each sample, qPCRs were performed in technical triplicate. Averages were normalized against input chromatin and further normalized against control-treated samples. Experiments were performed at least in biological triplicate and analyzed by Student’s t-test.

### siRNA-RNAiMAX transfection

Sequences of siRNAs targeting the *Foxg1* locus and their anti-GFP control are provided in Supplementary Table 1. For transfection, E16.5 mouse post-mitotic neurons were seeded in 12-well plates at about 3 × 10^5 cells/well in 600 μl Neurobasal A-based differentiative medium. At the same time of seeding, 15 pmol of each siRNA was complexed with 2 μl of Lipofectamine RNAiMAX reagent (Invitrogen) and transfected to the cells to a final 25 nM concentration, according to the manufacturer’s protocol. 24 h after transfection, medium was replaced and siRNAs were re-transfected as described above. RNA was extracted 48 h after the second transfection and analyzed by qRTPCR.

### siRNA-RVG-9dR transfection

The sequence of RVG-9dR peptide (synthesized by LifeTein) is reported in Supplementary Table 1. For RVG-9dR-mediated transfection, the different siRNA duplexes (100 pmol each) were incubated with RVG-9R peptide at a 1:10 molar ratio in 15 μl, for 15 min at room temperature. RNA-polypeptide binding was assessed on not-denaturing agarose gel by electrophoretic mobility shift assay. Next, the complexes were added to acutely dissociated E12.5 or E16.5 neural cells, plated in 24-well plates, at 1,5 × 10^5 cells/300 μl, or in 12-well plates, at 3 × 10^5 cells/600 μl, respectively. 24 h later, the transfection was repeated as described above. Cells were cultured for further 48h and, finally, RNA was extracted and examined by qRTPCR.

### Adeno-associated virus cloning and production

As for AAV production, genomic plasmids were obtained by transferring “Pgk1p-EGFP-pri-miRNA” modules from the corresponding lentiviral expressors into a scAAV2-type backbone [AAVscCB6(p1023)Q], upstream of a rabbit-polyA signal. Recombinant AAVs were packaged as previously described^69^.

### *In vivo* RNAa assays

P0 pups were anaesthetized on ice for 40–60 s. 3*10^10^ AAVs particles, mixed with 0.02% fast-green dye, were injected through the skull into the lateral ventricle, by free hands, using a sharp pulled micropipette (hole external diameter about 40 μm) with the help of light fibers. Animals were left to recover in a warm clean cage. Next they were transferred to their mother. 21 days later they were finally sacrificed. Brains were dissected from the skull, neocortices were homogenized and resuspended in TRIzol reagent (Ambion). Alternatively, for immunofluorescence, brains were fixed in fresh 4% PFA overnight at 4 °C. Next, they were cryoprotected overnight in 30% sucrose-1X PBS at 4 °C and finally frozen on dry ice in Killik (BioSigma).

### Immunofluorescence

Lentivirus transduced, floating neural precursor aggregates were gently trypsinized to single cells and left to attach 1 h at 37 °C to poly-L-lysine (200 μg*/*ml) coated SuperFrost Plus microscope slides (Menzel-Glaser). Here they were fixed by 4% PFA for 20 min at 4 °C, washed three times in 1X PBS and processed for immunofluorescence. Fixed-cryopreserved brains were sliced at 16 μm, tissue slices were allowed to dry at least one hour at RT and processed for immunofluorescence.

In all cases, immunofluorescence was performed as previously described^45^. The following primary antibodies were used: anti-Tubb3 (mouse clone Tuj1, Covance #MMS-435P, 1:1000); anti-GFP (chicken polyclonal, Abcam ab13970, 1:400); anti-Foxg1 (rabbit polyclonal, 1:200^41^). Secondary antibodies were conjugates of Alexa Fluor 488 and 594 (Invitrogen), used at 1:600. Cell nuclei were counterstained with DAPI (4′, 6′-diamidino-2-phenylindole).

Tubb3 immunofluorescences were photographed on a Nikon Eclipse TS100 fluorescence microscope equipped with a DS-2MBWC digital microscope camera with a 20X objective. Immunoprofiled brain sections were photographed on a Nikon TI-E microscope, equipped with 20X or 40X objectives and a Hamamatsu C4742–95 camera. All images were processed using Adobe 9.0.2 Photoshop 2 CS2 software and ImageJ.

### Statistical analysis

As for *in vitro* assays, each “biological replicate” included cells pooled from at least two independent wells*/*petri dishes. As for *in vivo* tests, each “biological replicate” corresponded to a single brain. Numbers of biological replicates analyzed in each experiment (*n*) are shown under the corresponding graphs. Each biological replicate was scored at least in technical triplicate.

Data were normalized as reported in figure legends and averaged. Variability was graphically shown by standard error of mean bars.

Statistical significance of results was evaluated by Student’s t-test (unpaired, one-tail) or ANCOVA. In case of multiple comparisons (Figs 1E,F and 4F,G), to make each dataset suitable for drawing reliable conclusions from its comprehensive evaluation, statistical results were further filtered by the Benjamini and Hochberg algorithm^70^. In such cases, the false discovery rate (FDR) was placed at <1/m, where m is the multiplicity of the comparison-set. Results of Benjamini and Hochberg filtering were summarized in Supplementary Table 2, panel 1E, 1F, 4F and 4 G datasets.

## Additional Information

**How to cite this article**: Fimiani, C. *et al*. RNA activation of haploinsufficient *Foxg1* gene in murine neocortex. *Sci. Rep.* **6**, 39311; doi: 10.1038/srep39311 (2016).

**Publisher's note:** Springer Nature remains neutral with regard to jurisdictional claims in published maps and institutional affiliations.

## Supplementary Material

Supplementary Materials
