# Supplementary material for: RNA activation of haploinsufficient Foxg1 gene in murine neocortex
Source: Sci Rep. 2016 Dec 20;6:39311. doi: 10.1038/srep39311 (PMC5172352; doi:10.1038/srep39311)
Supplement: Supplementary Materials [file srep39311-s1.pdf]

RNA activation of haploinsufficient *Foxg1* gene in murine neocortex

by Cristina Fimiani, Elisa Goina, Qin Su, Guangping Gao and Antonello Mallamaci

SUPPLEMENTARY MATERIALS

- **SUPPLEMENTARY FIGURES (1-4)**
- **SUPPLEMENTARY TABLES (1,2)**

SUPPLEMENTARY FIGURES

FIGURE S1

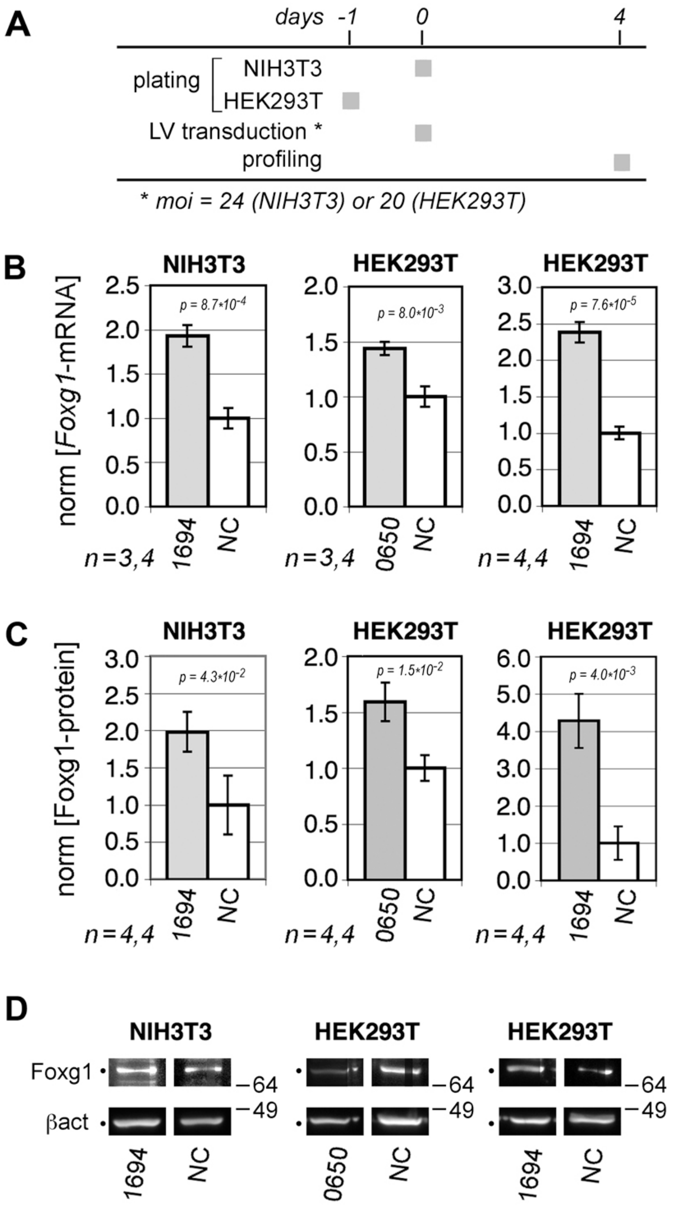

Figure S1. *Foxg1*-RNAa in NIH3T3 and HEK293T cells. (A) Temporal articulation of the assays. **(B)** qRTPCR evaluation of *Foxg1/FOXG1*-mRNA upon saRNA delivery. Values double normalized, against *Gapdh*/*GAPDH* and controls (NC). **(C)** Western blot evaluation of Foxg1/FOXG1-protein upon saRNA delivery. Values double normalized, against βActin/βACTIN and controls (NC). **(D)** Examples of western blots referred to in (C). Throughout the figure, "1694", "0650" and "NC" stand for miR-αFoxg1.1694, miR-αFoxg1.0650 and miR.NC,

respectively, Bars represent sem's. n = number of biological replicates. Evaluation of statistical significance by t-Student assay (one-tail, unpaired).

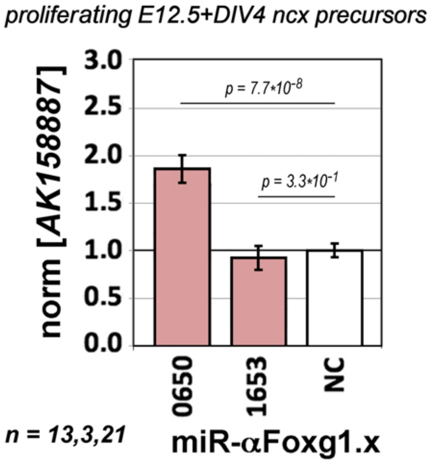

Figure S2. Impact of saRNAs on AK158887-ncRNA expression levels. AK158887-ncRNA levels in proliferating neocortical precursors manipulated as in Fig. 1B,C. qRTPCR assay performed on amplicon (2) of Fig. 4A. Values double normalized, against *Gapdh* and control (NC). E, embryonic day. DIV, days in vitro. Bars represent sem's. *n* = number of biological replicates. Evaluation of statistical significance by t-Student assay (one-tail, unpaired).

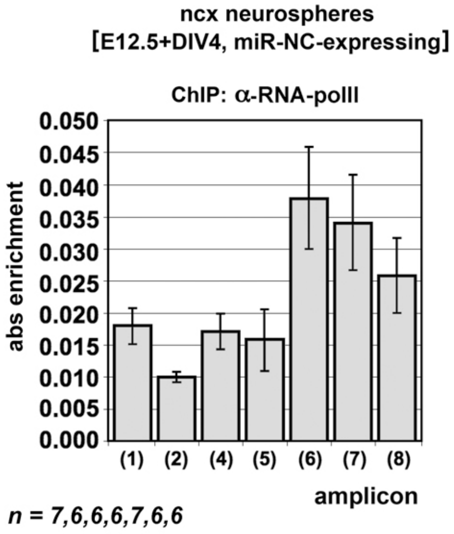

Figure S3. Absolute RNApolII enrichment at the *Foxg1* locus in cortico-cerebral precursors in control conditions. qPCR quantification of *Foxg1* chromatin enrichment, upon chromatin immunoprecipitation (ChIP) by antibodies against RNA polymerase II (α-RNA-polII). Evaluation performed in neocortical precursors challenged by miR-NC, according to the protocol shown in Figure 1B,C. Values normalized against input chromatin. Bars represent sem's. *n* = number of biological replicates.

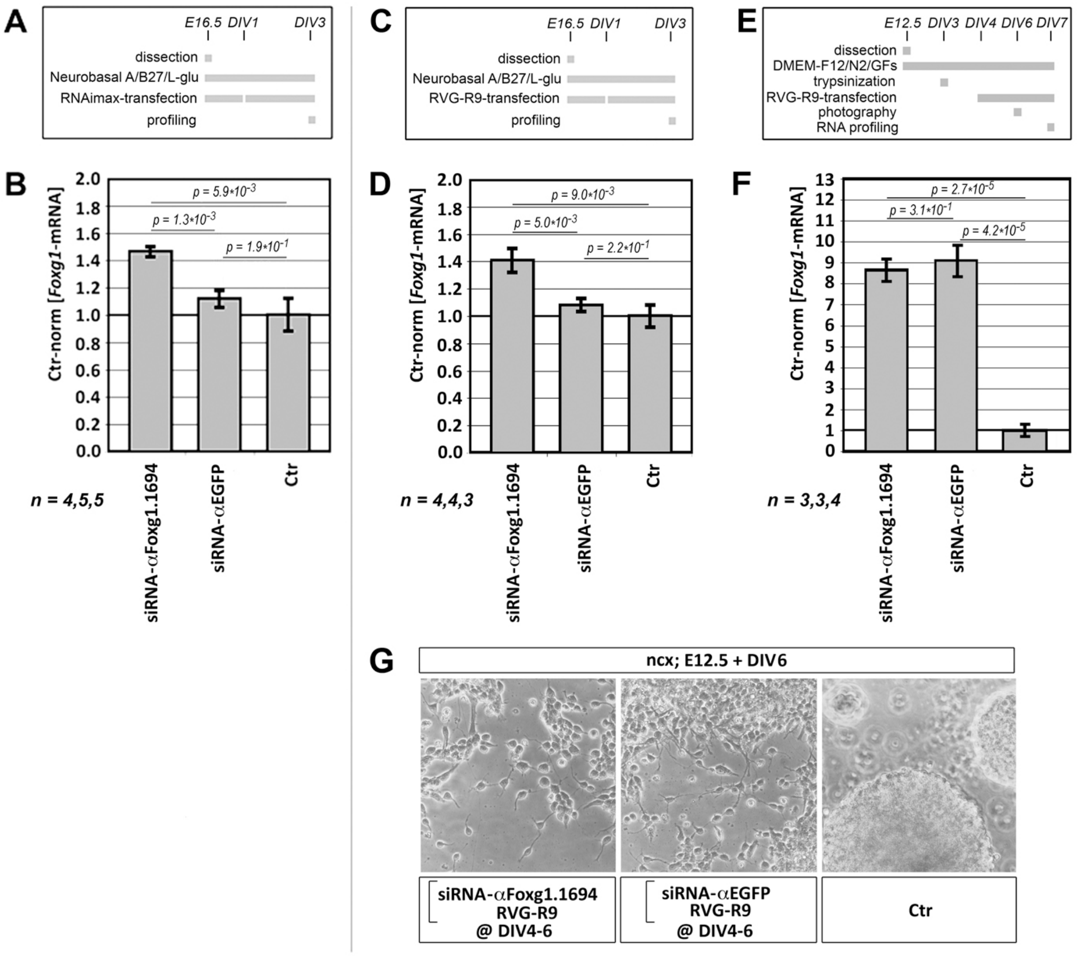

Figure S4. *Foxg1* stimulation in neocortical cultures treated by chemically synthesized saRNAs. (A,B) *Foxg1*-mRNA expression levels in neocortical differentiating cells treated by siRNA-αFoxg1.1694 or siRNA-αEGFP, according to the RNAimax-based protocol shown in (A). Ctr are untreatd controls. **(C,D)** *Foxg1*-mRNA expression levels in neocortical differentiating cells treated by siRNA-αFoxg1.1694 or siRNA-αEGFP, according to the RVG-9R-based protocol shown in (C). Ctr are untreatd controls. **(E,F)** *Foxg1*-mRNA expression levels in neocortical proliferating precursors treated by siRNA-αFoxg1.1694 or siRNA-αEGFP, according to the RVG-R9-based protocol shown in (E). Ctr are untreatd controls. (**G**) Examples of treated and control neural precursor cells referred to in (E,F). In (B,D,F), values double normalized, against *Gapdh* and control (Ctr). E, embryonic day. DIV, days in vitro. Bars represent sem's. *n* = number of biological replicates. Evaluation of statistical significance by t-Student assay (one-tail, unpaired).

SUPPLEMENTARY TABLE 1. Nucleic acid, morpholino and polypeptide sequences.

artificial miRNAs

name (miR-αFoxg.)	*sequence (5' - 3')*	*genomic target coord**	*Block-it score***	*off-target numbers ****	*human vs mouse target difference*****
0650	TAAACTTCCCAGTAAGAGGTC	chr12(+): 49,379,719-49,379,739	5/5	0,0	1 sub
0755	TACTGTTCTCGCAAACCTAAT	chr12(-): 49,379,746-49,379,766	NA	0,0	0
1653	TTGTGAAGTTGAAAGTCTCTG	chr12(+): 49,380,664-49,380,684	5/5	0,0	1 ins
1694	TTCAAATCCAATTTGCACCCA	chr12(-): 49,380,612-49,380,632	5/5	0,0	0
2273	TTGTATTCAAGGAACAACTCC	chr12(-): 49,381,265-49,381,285	4.5/5	0,0	4 sub
2764	AAATGATTCGGTGTAACCGGA	chr12(+): 49,381,769-49,381,789	5/5	0,0	0
3700	TTCCCAGGGATTGGAATTTAG	chr12(+): 49,382,712-49,382,732	4.5/5	0,0	0
3795	AGGATTTGTGATCTGTGTGCT	chr12(-): 49,382,686-49,382,706	NA	0,0	6 sub, 1 ins

* UCSC Genome Browser GRCm38/mm10

** scores according to the running version of Block-it software (09.16.2016); NA, not available [here, the candidate miRNA, selected by a past version of Block-it software (2012), could not be re-harvested by the current version of this software, as falling under the 3/5 threshold]

*** as evaluated by Blastn (NCBI, "*somehow similar sequence*") and Blat (UCSC), on mouse transcriptome and genome, respectively

**** sub, substitution; ins, insertion

GapmeRs

name	*sequence (5'-3')**
αAK158887-1.1 GapmeR	AGTTACTCTTTAGCGT
Negative Control A GapmeR	AACACGTCTATACGC

* underlined, LNA

Morpholinos

name	*sequence*
α-Ago1 morpholino	GTCCCGCTTCCATCCCATATACCCG
α-GFP morpholino	ACAGCTCCTCGCCCTTGCTCACCAT

siRNAs

<table>
<tr><th>name</th><th>sequence</th></tr>
<tr><td>siRNA-αFoxg1.0650</td><td>5' pUAAACUUCCCAGUAAGAGGUCGdTdT 3'
||||||||||||||||||||||
3' dTdTAUUUGAAGGGUCAUUCUCCAGCp 5'</td></tr>
<tr><td>siRNA-αFoxg1.1694</td><td>5' pUUCAAAUCCAAUUUGCACCCAGdTdT 3'
||||||||||||||||||||||
3' dTdTAAGUUUAGGUUAAACGUGGGUCp 5'</td></tr>
<tr><td>siRNA-αGFP</td><td>5' pGAACUUCAGGGUCAGCUUGdTdT 3'
|||||||||||||||||||
3' dTdTCUUGAAGUCCCAGUCGAACp 5'</td></tr>
</table>

polypeptide

name	*sequence (N-C)*
RVG-R9 peptide	YTIWMPENPRPGTPCDIFTNSRGKRASNGGGGRRRRRRRRR

DNA oligos

name	*sequence (5'-3')*	*qPCR destination*
mAK567Fw	GAGGCGACCGCTTCTGAACTGAGTAT	Fig.4A, amplicon (1)
mAK727Rev	TATAGACTTCCCTGAGAGCACAAACATCAA	
mAKintFw	TGCCATTATGTGTGAGTCTCTCTAGGG	Fig.4A, amplicon (2)
mAKintRev	GTAGCAAAGCTAGATCCACTAGCACCC	
mAK2110Fw	GTTGGCTCTAGGGAAGGCAGACTTCA	Fig.4A, amplicon (3)
mAK2225Rev	ATGCACAGGACGGTTTGTGACCTCTTG	
mAK2408Fw	CTGCTCGAATGAAGTGCGCTGTGTAAGC	Fig.4A, amplicon (4)
mAK2565Rev	CTCAGCTCTCATCCACTACCTACTCACTC	
mFoxg1.127Fw	TAGAAGCTGAAGAGGAGGTGGAGTGC	Fig.4A, amplicon (5)
mFoxg1.215Rev	CAGACCCAAACAGTCCCGAAATAAAGC	
mFoxCDS1085Fw	GACAAGAAGAACGGCAAGTACGAGAAGC	Fig.4A, amplicon (6)
mFoxCDS1144Rev	GAACTCATAGATGCCATTGAGCGTCAGG	
mFoxg1cdsFw	CGACCCTGCCCTGTGAGTCTTTAAG	Fig.4A, amplicon (7)
mFoxg1cdsRev	GGGTTGGAAGAAGACCCCTGATTTTGATG	
mFoxg1.2193Fw	TGGAGTGTCAGCGAGGTGCAATGTGG	Fig.1A;<br>Fig.4A, amplicon (8)
mFoxg1.2303Rev	TACTGCACACATGGAAATCTGGCAGCC	
mGapdhFw	ATCTTCTTGTGCAGTGCCAGCCTCGTC	murine *Gapdh* normalizer
mGapdhRev	GAACATGTAGACCATGTAGTTGAGGTCAATGAAGG	
hFoxg1For	TGGAGTGTCAGCGAGGTGCAATGTGG	Fig.S1 (HEK293T cells)
hFoxg1Rev	AATACTGCACACATGGAAATCTGGCGG	
hGapdhFor	CATCACCATCTTCCAGGAGCGAGATCC	human *Gapdh* normalizer
hGapdhRev	CAAATGAGCCCCAGCCTTCTCCATGG	

SUPPLEMENTARY TABLE 2. Primary data and statistical analysis, referred to in Figure panels 1E, 1F, 2C, 3C, 3E, 4B, 4C, 4E, 4F, 4G, 5D, 5E, S1B, S1C, S2, S3, S4B, S4D, S4F

PANEL 1E DATA

***Foxg1*-mRNA in E12.5-DIV4 ncx precursors**		number of amplicons		*Foxg1*-mRNA/ *Gapdh*-mRNA amplicon ratio	miRNA-NC-norm *Foxg1*-mRNA / *Gapdh*-mRNA amplicon ratio			p (t-test, vs miR-NC)	reject null hypothesis (Benjamini-Hochberg test, at FDR<1/m)
samples		*Foxg1*-mRNA	*Gapdh*-mRNA		value	average	sem		
miRNA-αFoxg1.0650	a	7.87E+04	2.19E+06	3.58E-02	2.22	2.88	0.34	1.8E-07	YES
	b	7.23E+04	1.89E+06	3.83E-02	2.38				
	c	1.32E+04	3.29E+05	4.01E-02	2.49				
	d	6.62E+03	9.24E+04	7.16E-02	4.44				
	e	6.53E+04	1.89E+06	3.45E-02	2.14				
	f	6.80E+04	1.83E+06	3.71E-02	2.30				
	g	1.90E+04	7.99E+05	2.38E-02	1.47				
	h	2.79E+04	5.67E+05	4.92E-02	3.05				
	i	1.52E+05	5.92E+06	2.57E-02	1.59				
	j	9.36E+04	2.07E+06	4.52E-02	2.80				
	k	2.08E+04	2.15E+05	9.65E-02	5.98				
	l	1.81E+05	2.88E+06	6.30E-02	3.91				
	m	5.63E+04	1.29E+06	4.38E-02	2.71				
miRNA-αFoxg1.0755	a	1.97E+04	9.31E+05	2.12E-02	1.31	2.50	0.38	3.9E-07	YES
	b	2.96E+03	8.31E+04	3.56E-02	2.21				
	c	6.51E+04	1.54E+06	4.22E-02	2.62				
	d	1.89E+04	4.27E+05	4.43E-02	2.75				
	e	5.01E+03	1.54E+05	3.25E-02	2.01				
	f	4.84E+04	7.35E+05	6.59E-02	4.09				
miRNA-αFoxg1.1694	a	3.77E+04	1.96E+06	1.93E-02	1.20	1.94	0.20	9.5E-07	YES
	b	1.05E+05	2.46E+06	4.26E-02	2.64				
	c	3.58E+04	1.13E+06	3.18E-02	1.97				
	d	3.02E+04	8.07E+05	3.74E-02	2.32				
	e	1.13E+05	3.58E+06	3.15E-02	1.95				
	f	2.72E+04	1.73E+06	1.58E-02	0.98				
	g	5.00E+04	1.12E+06	4.45E-02	2.76				
	h	1.78E+05	5.84E+06	3.05E-02	1.89				
	i	9.52E+03	3.39E+05	2.81E-02	1.74				
miRNA-αFoxg1.1653	a	6.75E+04	2.16E+06	3.13E-02	1.94	1.54	0.25	1.4E-03	YES
	b	6.10E+03	2.36E+05	2.59E-02	1.61				
	c	2.21E+04	1.27E+06	1.73E-02	1.08				
miRNA-αFoxg1.2273	a	3.09E+04	1.67E+06	1.85E-02	1.15	1.38	0.11	2.8E-03	YES
	b	4.52E+04	1.82E+06	2.47E-02	1.53				
	c	1.81E+04	9.04E+05	2.01E-02	1.24				
	d	2.48E+04	9.54E+05	2.60E-02	1.61				

miRNA-αFoxg1.2764	a	7.58E+03	5.09E+05	1.49E-02	0.92	1.28	0.24	4.6E-02	YES
	b	1.91E+04	1.01E+06	1.89E-02	1.17				
	c	5.59E+04	2.00E+06	2.80E-02	1.73				
miRNA-αFoxg1.3795	a	3.90E+04	1.23E+06	3.16E-02	1.96	1.59	0.20	4.1E-04	YES
	b	2.18E+04	1.04E+06	2.10E-02	1.30				
	c	1.68E+05	6.96E+06	2.41E-02	1.50				
miRNA-αFoxg1.3700	a	1.90E+04	6.38E+05	2.98E-02	1.85	1.51	0.12	6.5E-05	YES
	b	3.33E+04	1.21E+06	2.76E-02	1.71				
	c	3.30E+04	1.48E+06	2.23E-02	1.38				
	d	1.16E+05	4.22E+06	2.74E-02	1.70				
	e	4.40E+04	2.43E+06	1.81E-02	1.12				
	f	2.06E+04	9.73E+05	2.12E-02	1.32				
miR-NC	a	3.75E+04	1.81E+06	2.07E-02	1.28	1.00	0.05	==	==
	b	1.56E+04	1.23E+06	1.27E-02	0.79				
	c	2.01E+04	1.34E+06	1.50E-02	0.93				
	d	3.10E+04	1.47E+06	2.11E-02	1.31				
	e	6.71E+03	5.14E+05	1.31E-02	0.81				
	f	2.90E+04	2.04E+06	1.42E-02	0.88				
	g	2.00E+04	1.12E+06	1.78E-02	1.10				
	h	2.50E+04	1.73E+06	1.44E-02	0.90				
	i	1.67E+04	1.88E+06	8.88E-03	0.55				
	j	3.25E+03	2.21E+05	1.47E-02	0.91				
	k	2.54E+04	1.03E+06	2.48E-02	1.54				
	l	1.93E+03	9.60E+04	2.01E-02	1.25				
	m	1.65E+03	1.30E+05	1.27E-02	0.79				
	n	3.84E+04	2.46E+06	1.56E-02	0.97				
	o	2.74E+04	1.61E+06	1.70E-02	1.05				
	p	1.46E+04	9.56E+05	1.53E-02	0.95				
	q	3.87E+04	2.17E+06	1.78E-02	1.11				
	r	3.68E+03	2.34E+05	1.57E-02	0.98				
	t	8.51E+02	5.75E+04	1.48E-02	0.92				

PANEL 1F DATA

Foxg1-mRNA in differentiating E16.5-DIV4 ncx cells		number of amplicons		amplicon ratio	miRNA-NC-norm *Foxg1*-mRNA / *Gapdh*-mRNA amplicon ratio			p (t-test, vs miR-NC)	reject null hypothesis (Benjamini -Hochberg test, at FDR<1/m)
samples		*Foxg1-mRNA*	*Gapdh-mRNA*	*Foxg1-mRNA/ Gapdh-mRNA*	values	average	sem		
miR-aFoxg1.0650	a	8.27E+02	9.57E+03	8.65E-02	1.62	0.89	0.23	3.3E-01	NOT
	b	2.65E+03	5.51E+04	4.80E-02	0.90				
	c	1.69E+03	7.48E+04	2.26E-02	0.42				
	d	3.26E+03	5.49E+04	5.94E-02	1.11				
	e	2.52E+03	1.14E+05	2.21E-02	0.41				
miR-aFoxg1.0755	a	2.35E+03	3.73E+04	6.31E-02	1.18	1.08	0.16	3.6E-01	NOT
	b	9.22E+02	1.96E+04	4.71E-02	0.88				
	c	2.70E+03	5.23E+04	5.15E-02	0.97				
	d	6.56E+02	1.69E+04	3.88E-02	0.73				
	e	8.72E+03	9.91E+04	8.80E-02	1.65				
miR-aFoxg1.1694	a	5.52E+03	5.36E+04	1.03E-01	1.93	1.56	0.11	5.1E-03	YES
	b	3.17E+03	4.70E+04	6.76E-02	1.27				
	c	7.49E+03	9.65E+04	7.76E-02	1.45				
	d	2.34E+04	2.42E+05	9.65E-02	1.81				
	e	2.25E+03	2.74E+04	8.21E-02	1.54				
	f	5.60E+03	7.80E+04	7.18E-02	1.35				
miR-aFoxg1.3700	a	2.75E+03	3.67E+04	7.49E-02	1.40	1.07	0.20	3.9E-01	NOT
	b	3.78E+02	5.03E+03	7.52E-02	1.41				
	c	3.79E+03	9.04E+04	4.19E-02	0.79				
	d	1.88E+03	5.18E+04	3.62E-02	0.68				
miR-NC	a	5.01E+03	1.05E+05	4.75E-02	0.89	1.00	0.13	==	==
	b	2.88E+03	4.63E+04	6.23E-02	1.17				
	c	3.04E+03	6.06E+04	5.02E-02	0.94				
	d	5.73E+03	7.24E+04	7.91E-02	1.48				
	e	2.14E+03	4.77E+04	4.49E-02	0.84				
	f	1.33E+03	3.71E+04	3.59E-02	0.67				
	g	3.60E+03	6.18E+04	5.82E-02	1.09				
	h	4.40E+03	8.92E+04	4.93E-02	0.92				
	i	1.70E+03	7.69E+04	2.22E-02	0.42				
	j	5.26E+03	5.10E+04	1.03E-01	1.93				
	k	3.35E+02	9.61E+03	3.49E-02	0.65				

PANEL 2C DATA

**Frequencies of Tubb3+ newborn neurons in cultures manipulated by *Foxg1*-RNAa**			Tubb3+ cells	Dapi+ cells	Tubb3+ / Dapi+ cell ratio	miRNA-NC-norm Tubb3+ / Dapi+ cell ratio			p (t-test, vs-miRNA-NC)
samples						value	average	sem	
1	miRNA-αFoxg1. 0650	a	123	960	0.1281	0.4702	0.4575	0.0144	3.3E-06
		b	172	1487	0.1157	0.4245			
		c	164	1260	0.1302	0.4777			
2	miRNA-αFoxg1. 1694	a	120	868	0.1382	0.5074	0.5415	0.0148	7,3E-06
		b	148	977	0.1515	0.5560			
		c	150	981	0.1529	0.5612			
3	miRNA-NC	a	269	997	0.2698	0.9902	1.0000	0.0052	==
		b	377	1385	0.2722	0.9990			
		c	355	1289	0.2754	1.0108			

PANEL 3C DATA

***Foxg1*-mRNA modulation by RNAa in ncx and me/rh/c precursors**				number of amplicons		*Foxg1*-mRNA / *Gapdh*-mRNA amplicon ratio			p (t-test)
exp #	precursors origin	miRNA	sam ple	*Foxg1*-mRNA	*Gapdh*-mRNA	value	average	sem	
1	me/rh/c	miRNA-αFoxg1. 0650	a	8.10E+01	2.77E+06	2.92E-05	2.98E-05	3.48E-06	*1 vs 3* 1.1E-02
			b	4.21E+01	1.74E+06	2.42E-05			
			c	5.86E+01	1.62E+06	3.61E-05			
2		miRNA-αFoxg1. 1694	a	6.44E+00	1.62E+06	3.99E-06	1.32E-05	9.38E-06	*2 vs 3* 3.8E-01
			b	7.11E+00	1.93E+06	3.68E-06			
			c	1.77E+01	5.54E+05	3.20E-05			
3		miRNA-NC	a	4.06E+01	2.76E+06	1.47E-05	9.85E-06	4.34E-06	*1 vs 6* 4.5E-03
			b	2.42E+01	1.77E+06	1.36E-05			
			c	3.99E+00	3.31E+06	1.21E-06			*2 vs 6* 4.5E-03
4	ncx	miRNA-αFoxg1. 0650	a	1.90E+04	7.14E+05	2.66E-02	2.52E-02	5.39E-03	
			b	2.01E+04	5.96E+05	3.37E-02			*3 vs 6* 4.5E-03
			c	1.40E+04	9.19E+05	1.53E-02			
5		miRNA-αFoxg1. 1694	a	2.40E+04	9.37E+05	2.56E-02	2.01E-02	3.42E-03	*4 vs 6* 3.1E-02
			b	6.44E+03	4.64E+05	1.39E-02			
			c	9.02E+03	4.34E+05	2.08E-02			*5 vs 6* 3.6E-02
6		miRNA-NC	a	9.95E+03	8.94E+05	1.11E-02	1.03E-02	2.16E-03	
			b	6.13E+03	9.93E+05	6.17E-03			
			c	7.56E+03	5.60E+05	1.35E-02			

PANEL 3E DATA

***Foxg1*-mRNA modulation by RNAa and extracellular K$^+$**				number of amplicons		*Foxg1*-mRNA / *Gapdh*-mRNA amplicon ratio	(miRNA-NC & $t_{(K^+)}$=0h)-norm *Foxg1*-mRNA / *Gapdh*-mRNA amplicon ratio			p (t-test)
exp #	miRNA	$t_{(25mMK+)}$	sample	*Foxg1*-mRNA	*Gapdh*-mRNA		value	average	sem	
1	miRNA-αFoxg1.1694	0h	a	3.08E+04	4.48E+05	6.87E-02	1.87	1.91	0.10	
			b	1.15E+03	1.44E+04	7.97E-02	2.17			
			c	2.17E+04	3.51E+05	6.19E-02	1.69			
			d	7.97E+03	1.13E+05	7.06E-02	1.93			*1 vs 4* 1.7E-03
2		3h	a	6.84E+04	6.03E+05	1.13E-01	3.09	2.71	0.20	
			b	3.29E+04	3.71E+05	8.87E-02	2.42			*2 vs 5* 1.6E-02
			c	7.51E+04	7.81E+05	9.62E-02	2.62			
3		6h	a	2.37E+04	2.00E+05	1.19E-01	3.24	3.14	0.11	*3 vs 6* 1.1E-02
			b	3.10E+04	2.58E+05	1.20E-01	3.27			
			c	3.63E+03	3.40E+04	1.07E-01	2.91			*2 vs 1* 5.8E-03
4	miRNA-NC	0h	a	8.10E+02	3.43E+04	2.36E-02	0.64	1.00	0.17	
			b	2.36E+03	6.05E+04	3.89E-02	1.06			
			c	6.45E+03	2.03E+05	3.17E-02	0.86			*3 vs 1* 2.0E-04
			d	1.34E+04	2.55E+05	5.25E-02	1.43			
5		3h	a	9.88E+03	1.24E+05	8.00E-02	2.18	1.95	0.13	*5 vs 4* 4.1E-03
			b	1.50E+04	2.35E+05	6.39E-02	1.74			
			c	3.07E+04	4.31E+05	7.12E-02	1.94			*6 vs 4* 3.1E-03
6		6h	a	3.14E+04	3.37E+05	9.32E-02	2.54	2.22	0.22	
			b	3.26E+04	3.80E+05	8.58E-02	2.34			
			c	1.06E+04	1.61E+05	6.60E-02	1.80			

PANEL 4B DATA

***AK15887*-ncRNA in NIH/3T3 cells**			number of amplicons		*AK158887*-ncRNA / *Gapdh*-mRNA amplicon ratio	(miRNA-NC & gapmer-NC)-norm *AK158887*-ncRNA / *Gapdh*-mRNA amplicon ratio			p (t-test)
samples			*AK158887* -ncRNA	*Gapdh*-mRNA		value	aver -age	sem	
1	miRNA- αFoxg1.0650 + gapmer- aAK158887-1.1	a	1.40E+03	2.29E+06	6.10E-04	0.52	0.58	0.03	
		b	2.60E+03	3.41E+06	7.63E-04	0.65			
		c	2.36E+03	3.34E+06	7.08E-04	0.61			
		d	1.79E+03	2.88E+06	6.21E-04	0.53			
2	miRNA- αFoxg1.0650 + gapmer-NC	a	3.44E+03	2.98E+06	1.15E-03	0.99	0.97	0.01	*1-vs-2* 1.0E-05
		b	3.98E+03	3.62E+06	1.10E-03	0.94			
		c	3.57E+03	3.14E+06	1.14E-03	0.97			
		d	3.48E+03	3.05E+06	1.14E-03	0.98			1-*vs-3* 2.3E-01
3	miRNA-NC + gapmer- αAK158887-1.1	a	2.32E+03	3.41E+06	6.79E-04	0.58	0.61	0.02	
		b	1.76E+03	2.68E+06	6.58E-04	0.56			
		c	2.36E+03	3.07E+06	7.68E-04	0.66			*2-vs-4* 2.5E-01
		d	2.32E+03	3.16E+06	7.34E-04	0.63			
4	miRNA-NC + gapmer-NC	a	2.93E+03	2.46E+06	1.19E-03	1.02	1.00	0.04	*3-vs-4* 8.4E-05
		b	4.23E+03	3.55E+06	1.19E-03	1.02			
		c	3.79E+03	2.99E+06	1.27E-03	1.08			
		d	3.80E+03	3.68E+06	1.03E-03	0.88			

***Foxg1*-mRNA in NIH/3T3 cells**			number of amplicons		*Foxg1*-mRNA / *Gapdh*-mRNA amplicon ratio	(miRNA-NC & gapmer-NC)-norm *AK158887*-ncRNA / *Gapdh*-mRNA amplicon ratio			p (t-test)
samples			*Foxg1*-mRNA	*Gapdh*-mRNA		value	aver -age	sem	
1	miRNA- αFoxg1.0650 + gapmer- αAK158887-1.1	a	2.29E+06	8.71E+04	3.81E-02	1.08	1.01	0.06	*1-vs-2* 2.6E-03
		b	3.41E+06	1.28E+05	3.76E-02	1.06			
		c	3.34E+06	1.27E+05	3.81E-02	1.08			
		d	2.88E+06	8.47E+04	2.95E-02	0.83			*1-vs-4* 4.3E-01
2	miRNA- αFoxg1.0650 + gapmer-NC	a	2.98E+06	1.49E+05	5.01E-02	1.42	1.32	0.04	
		b	3.62E+06	1.71E+05	4.73E-02	1.34			
		c	3.14E+06	1.37E+05	4.36E-02	1.24			*2-vs-4* 1.2E-03
		d	3.05E+06	1.39E+05	4.56E-02	1.29			
3	miRNA-NC + gapmer- αAK158887-1.1	a	3.41E+06	1.18E+05	3.45E-02	0.98	0.92	0.06	*3-vs-4* 1.7E-01
		b	2.68E+06	7.81E+04	2.92E-02	0.83			

		c	3.07E+06	1.15E+05	3.75E-02	1.06			
		d	3.16E+06	8.91E+04	2.82E-02	0.80			
4	miRNA-NC + gapmer-NC	a	2.46E+06	8.99E+04	3.66E-02	1.04	1.00	0.05	
		b	3.55E+06	1.26E+05	3.55E-02	1.01			
		c	2.99E+06	1.16E+05	3.87E-02	1.10			
		d	3.68E+06	1.12E+05	3.03E-02	0.86			

PANEL 4C DATA

**Ago2 enrichment at *Foxg1* amplicon (3)**		number of amplicons		αAgo2-IP / input amplicon ratio	miRNA-NC-norm αAgo2-IP / input amplicon ratio			p (t-test)
sample		aAgo2-IP	input		value	average	sem	
miRNA-αFoxg1.0650	a	4.46E+02	9.68E+04	4.61E-03	1.21	1.38	0.20	1.8E-01
	b	3.75E+02	8.99E+04	4.17E-03	1.09			
	c	7.67E+02	1.09E+05	7.01E-03	1.84			
miRNA-NC	a	6.74E+02	1.30E+05	5.20E-03	1.36	1.00	0.30	
	b	4.07E+02	8.75E+04	4.65E-03	1.22			
	c	2.52E+02	1.60E+05	1.58E-03	0.41			

**Ago1 enrichment at *Foxg1* amplicon (3)**		number of amplicons		αAgo1-IP / input amplicon ratio	miRNA-NC-norm αAgo1-IP / input amplicon ratio			p (t-test)
samples		aAgo1-IP	input		values	average	sem	
miRNA-αFoxg1.0650	a	1.91E+02	2.34E+04	8.15E-03	1.53	1.78	0.26	6.3E-02
	b	2.19E+02	2.67E+04	8.22E-03	1.55			
	c	2.47E+02	3.13E+04	7.90E-03	1.49			
	d	2.78E+02	2.03E+04	1.37E-02	2.57			
miRNA-NC	a	2.86E+02	3.35E+04	8.53E-03	1.60	1.00	0.35	
	b	1.25E+02	2.36E+04	5.29E-03	1.00			
	c	3.83E+01	1.80E+04	2.12E-03	0.40			

PANEL 4D DATA

**Ago2 enrichment at *Foxg1* amplicon (4)**		number of amplicons		αAgo2-IP / input amplicon ratio	miRNA-NC-norm αAgo2-IP / input amplicon ratio			p (t-test)
samples		aAgo2-IP	input		value	average	sem	
miRNA-αFoxg1.1694	a	2.61E+02	9.12E+04	2.86E-03	0.81	0.89	0.19	3.3E-01
	b	2.37E+02	1.16E+05	2.05E-03	0.58			
	c	2.91E+02	1.15E+05	2.52E-03	0.72			
	d	4.45E+02	8.70E+04	5.11E-03	1.45			
miRNA-NC	a	3.64E+02	1.51E+05	2.41E-03	0.68	1.00	0.14	
	b	4.10E+02	1.19E+05	3.45E-03	0.98			
	c	5.16E+02	1.07E+05	4.81E-03	1.36			
	d	2.38E+02	6.90E+04	3.45E-03	0.98			

**Ago1 enrichment at *Foxg1* amplicon (4)**		number of amplicons		αAgo1-IP / input amplicon ratio	miRNA-NC-norm αAgo1-IP / input amplicon ratio			p (t-test)
samples		aAgo1-IP	input		value	average	sem	
miRNA-αFoxg1.1694	a	1.10E+03	6.07E+04	1.82E-02	2.40	2.12	0.23	4.8E-03
	b	1.18E+03	9.41E+04	1.25E-02	1.66			
	c	1.16E+03	6.68E+04	1.74E-02	2.30			
miRNA-NC	a	1.02E+03	1.38E+05	7.38E-03	0.98	1.00	0.06	
	b	1.07E+03	1.27E+05	8.39E-03	1.11			
	c	4.43E+02	6.40E+04	6.91E-03	0.91			

PANEL 4E DATA

***Foxg1*-mRNA in NIH/3T3 cells**			number of amplicons		*Foxg1-mRNA / Gapdh-mRNA* amplicon ratio	(miRNA-NC & morpholino-NC)-norm *Foxg1*-RNA /*Gapdh*-mRNA ratio			p (t-test)
sample			*Gapdh-mRNA*	*Foxg1-mRNA*		value	aver-age	sem	
1	miRNA-αFoxg1.1694 + morpholino-αAgo1	a	3.60E+05	3.49E+03	9.67E-03	1.16	1.00	0.07	*1-vs-2* 2.0E-03<br>*1-vs-4* 5.0E-01<br>*2-vs-4* 9.0E-04<br>*3-vs-4* 1.7E-01
		b	2.74E+05	2.21E+03	8.05E-03	0.96			
		c	1.78E+05	1.30E+03	7.34E-03	0.88			
2	miRNA-αFoxg1.1694 + morpholino-NC	a	6.24E+05	9.19E+03	1.47E-02	1.76	1.90	0.11	
		b	2.19E+04	3.95E+02	1.80E-02	2.16			
		c	3.70E+04	5.53E+02	1.49E-02	1.79			
3	miRNA-NC + morpholino-αAgo1	a	1.92E+05	1.54E+03	8.01E-03	0.96	1.14	0.09	
		b	5.52E+05	6.03E+03	1.09E-02	1.31			
		c	4.06E+05	3.91E+03	9.62E-03	1.15			
4	miRNA-NC + morpholino-NC	a	2.19E+05	2.14E+03	9.76E-03	1.17	1.00	0.09	
		b	3.71E+05	3.49E+03	9.39E-03	1.12			
		c	3.24E+05	2.19E+03	6.75E-03	0.81			
		d	3.80E+05	2.86E+03	7.53E-03	0.90			

PANEL 4F DATA

RNApolII enrichment at the *Foxg1* locus			number of amplicons		aRNApolII-IP / input amplicon ratio			miRNA-NC-norm aRNApolII-IP / input amplicon ratio			p (t-test)	reject the null hypothesis (Benjamini -Hochberg test, at FDR<1/m)
ampl -icon #	miR	sam ple	aRNApolII -IP	input	value	aver- age	sem	value	aver- age	sem		
(1)	miRNA-αFoxg1. 0650	a	2.74E+03	6.98E+04	0.039	0.036	0.003	1.52	1.38	0.12	7.1E-02	NOT
		b	2.64E+03	8.92E+04	0.030			1.14				
		c	2.33E+03	6.09E+04	0.038			1.47				
	miRNA-NC	a	2.65E+03	9.73E+04	0.027	0.026	0.004	1.05	1.00	0.17		
		b	2.12E+03	6.46E+04	0.033			1.26				
		c	1.85E+03	1.04E+05	0.018			0.69				
(2)	miRNA-αFoxg1. 0650	a	1.59E+03	4.69E+04	0.034	0.033	0.004	2.83	2.78	0.31	2.6E-03	YES
		b	1.35E+03	5.06E+04	0.027			2.22				
		c	1.10E+03	2.80E+04	0.039			3.29				
	miRNA-NC	a	1.06E+03	9.39E+04	0.011	0.012	0.001	0.94	1.00	0.09		
		b	7.00E+02	4.98E+04	0.014			1.18				
		c	7.33E+02	6.93E+04	0.011			0.88				
(4)	miRNA-αFoxg1. 0650	a	1.58E+03	4.02E+04	0.039	0.035	0.002	1.88	1.67	0.11	1.7E-02	YES
		b	1.54E+03	4.83E+04	0.032			1.52				
		c	1.23E+03	3.66E+04	0.034			1.60				
	miRNA-NC	a	1.36E+03	6.60E+04	0.021	0.021	0.004	0.98	1.00	0.18		
		b	1.12E+03	4.07E+04	0.028			1.32				
		c	1.00E+03	6.86E+04	0.015			0.70				
(5)	miRNA-αFoxg1. 0650	a	1.02E+03	2.24E+04	0.045	0.046	0.001	1.93	1.97	0.03	1.2E-02	YES
		b	1.28E+03	2.78E+04	0.046			1.96				
		c	8.24E+02	1.72E+04	0.048			2.03				
	miRNA-NC	a	8.72E+02	4.80E+04	0.018	0.024	0.006	0.77	1.00	0.28		
		b	8.86E+02	2.43E+04	0.036			1.55				
		c	5.48E+02	3.42E+04	0.016			0.68				
(6)	miRNA-αFoxg1. 0650	a	5.00E+03	5.03E+04	0.099	0.083	0.009	1.80	1.50	0.16	4.9E-02	YES
		b	4.40E+03	6.29E+04	0.070			1.27				
		c	3.16E+03	3.99E+04	0.079			1.44				
	miRNA-NC	a	4.53E+03	7.31E+04	0.062	0.055	0.010	1.13	1.00	0.17		
		b	3.78E+03	5.63E+04	0.067			1.22				
		c	2.48E+03	6.87E+04	0.036			0.66				
(7)	miRNA-αFoxg1. 0650	a	3.84E+03	4.00E+04	0.096	0.083	0.007	2.18	1.90	0.16	3.0E-02	YES
		b	3.98E+03	4.83E+04	0.082			1.87				

		c	2.92E+03	4.05E+04	0.072			1.64				
	miRNA-NC	a	3.08E+03	6.84E+04	0.045	0.044	0.014	1.02	1.00	0.31		
		b	2.66E+03	3.99E+04	0.067			1.52				
		c	1.38E+03	6.88E+04	0.020			0.46				
(8)	miRNA-αFoxg1.0650	a	2.23E+04	2.62E+05	0.085	0.075	0.005	2.31	2.04	0.15	1.1E-02	YES
		b	2.44E+04	3.28E+05	0.074			2.02				
		c	1.65E+04	2.49E+05	0.066			1.80				
	miRNA-NC	a	1.97E+04	4.65E+05	0.042	0.037	0.009	1.15	1.00	0.24		
		b	1.46E+04	2.99E+05	0.049			1.33				
		c	8.53E+03	4.42E+05	0.019			0.52				

PANEL 4G DATA

RNAa-dependent RNApolII enrichment at the *Foxg1* locus			number of amplicons		αRNApolII-IP / input amplicon ratio			miRNA-NC-norm aRNApolII-IP/input amplicon ratio			p (t-test)	reject the null hypothesis (Benjamini -Hochberg test, at FDR<1/m)
ampl -icon	miR	samp le	αRNApolII -IP	input	value	aver-age	sem	value	aver -age	sem		
(1)	miRNA-αFoxg1. 1694	a	1.13E+03	3.03E+04	0.037	0.022	0.007	3.07	1.77	0.58	9.8E-02	NOT
		b	1.07E+03	1.01E+05	0.011			0.87				
		c	1.55E+03	1.14E+05	0.014			1.12				
		d	9.92E+02	4.02E+04	0.025			2.03				
	miRNA-NC	a	1.54E+03	8.86E+04	0.017	0.012	0.003	1.43	1.00	0.21		
		b	7.52E+02	1.07E+05	0.007			0.58				
		c	1.32E+03	9.72E+04	0.014			1.12				
		d	1.19E+03	1.11E+05	0.011			0.88				
(2)	miRNA-αFoxg1. 1694	a	1.08E+03	2.91E+04	0.037	0.025	0.008	4.63	3.12	0.95	4.8E-02	YES
		b	1.34E+03	1.21E+05	0.011			1.38				
		c	1.19E+03	4.39E+04	0.027			3.36				
	miRNA-NC	a	4.70E+02	1.12E+05	0.004	0.008	0.002	0.52	1.00	0.24		
		b	1.08E+03	1.06E+05	0.010			1.27				
		c	1.05E+03	1.07E+05	0.010			1.21				
(4)	miRNA-αFoxg1. 1694	a	1.74E+03	4.67E+04	0.037	0.028	0.006	3.21	2.36	0.55	4.1E-02	YES
		b	2.44E+03	1.57E+05	0.016			1.33				
		c	1.73E+03	5.88E+04	0.030			2.53				
	miRNA-NC	a	1.97E+03	1.22E+05	0.016	0.012	0.002	1.38	1.00	0.21		
		b	1.24E+03	1.59E+05	0.008			0.67				
		c	1.56E+03	1.40E+05	0.011			0.95				
(5)	miRNA-αFoxg1. 1694	a	1.84E+02	4.02E+03	0.046	0.023	0.012	5.92	2.96	1.49	1.3E-01	NOT
		b	1.48E+02	1.64E+04	0.009			1.17				
		c	4.66E+01	3.35E+03	0.014			1.80				
	miRNA-NC	a	1.15E+02	1.74E+04	0.007	0.008	0.001	0.86	1.00	0.10		
		b	1.20E+02	1.30E+04	0.009			1.20				
		c	6.94E+01	9.51E+03	0.007			0.95				
(6)	miRNA-αFoxg1. 1694	a	4.48E+02	5.01E+03	0.089	0.065	0.018	3.53	2.55	0.69	3.2E-02	NOT
		b	7.11E+02	2.32E+04	0.031			1.21				
		c	5.41E+02	7.36E+03	0.073			2.90				
	miRNA-NC	a	6.59E+02	1.56E+04	0.042	0.025	0.007	1.66	1.00	0.29		
		b	2.79E+02	2.54E+04	0.011			0.43				
		c	5.66E+02	2.18E+04	0.026			1.03				
		d	5.07E+02	2.29E+04	0.022			0.87				
(7)	miRNA-αFoxg1. 1694	a	8.98E+03	1.20E+05	0.075	0.053	0.013	3.21	2.26	0.55	4.7E-02	YES
		b	1.30E+04	4.21E+05	0.031			1.32				
		c	1.00E+04	1.91E+05	0.053			2.26				
	miRNA-NC	a	5.59E+03	3.93E+05	0.014	0.023	0.005	0.61	1.00	0.20		
		b	9.47E+03	3.49E+05	0.027			1.16				
		c	1.15E+04	4.00E+05	0.029			1.23				
(8)	miRNA-αFoxg1. 1694	a	4.76E+03	9.34E+04	0.051	0.036	0.009	3.28	2.30	0.60	4.9E-02	YES
		b	5.74E+03	3.03E+05	0.019			1.22				

		c	4.56E+03	1.22E+05	0.037			2.40				
	miRNA-NC	a	3.52E+03	2.76E+05	0.013	0.016	0.001	0.82	1.00	0.09		
		b	4.44E+03	2.65E+05	0.017			1.08				
		c	4.53E+03	2.63E+05	0.017			1.10				

PANEL 5D DATA

Foxg1 expression levels in neocortices manipulated by in vivo RNAa		number of amplicons		*Foxg1*-mRNA / *Gapdh*-mRNA amplicon ratio	miRNA-NC-norm *Foxg1*-mRNA / *Gapdh*-mRNA ratio			p (t-test)
brains		*Foxg1*-mRNA	*Gapdh*-mRNA		value	aver-age	sem	
miRNA-αFoxg1.1694	a	2.79E+05	3.61E+06	7.72E-02	1.14	1.66	0.30	2.0E-02
	b	3.31E+05	3.79E+06	8.73E-02	1.29			
	c	6.51E+04	8.62E+05	7.55E-02	1.12			
	d	1.92E+05	1.28E+06	1.50E-01	2.23			
	e	5.58E+05	3.26E+06	1.71E-01	2.54			
miRNA-NC	a	1.58E+05	2.35E+06	6.72E-02	1.01	1.00	0.02	
	b	1.00E+05	1.56E+06	6.45E-02	0.96			
	c	1.12E+05	1.59E+06	7.05E-02	1.04			
	d	1.27E+05	1.85E+06	6.85E-02	1.02			
	e	8.18E+04	1.30E+06	6.29E-02	0.93			
	f	1.73E+05	2.44E+06	7.10E-02	1.05			

PANEL 5E DATA

**Frequencies of Foxg1+ neocortical cells transduced by miRNA-NC/Egfp-expressing AAVs**			Egfp+Foxg1+ / Foxg1+ cell ratio		
brains	Egfp+ Foxg1+ cells	Foxg1+ cells	value	average	sem
a	376	1976	0.1903	0.1657	0.0107
b	261	1642	0.1590		
c	193	1379	0.1400		
d	346	1994	0.1735		

PANEL S1B DATA

**RNAa in cell lines. *Foxg1*-mRNA levels**			number of amplicons		*Foxg1*-mRNA / *Gapdh*-*mRNA* amplicon ratio	miR-NC-norm *Foxg1*-mRNA / *Gapdh*-mRNA amplicon ratio			p-value (t-test)
cell line	sample		*Foxg1*-mRNA	*Gapdh*-mRNA		value	aver-age	sem	
NIH/3T3	miRNA-αFoxg1.1694	1	6.24E+05	9.19E+03	1.47E-02	1.76	1.90	0.13	8.7E-04
		2	2.19E+04	3.95E+02	1.80E-02	2.16			
		3	3.70E+04	5.53E+02	1.49E-02	1.79			
	miRNA.NC	1	2.19E+05	2.14E+03	9.76E-03	1.17	1.00	0.09	
		2	3.71E+05	3.49E+03	9.39E-03	1.12			
		3	3.24E+05	2.19E+03	6.75E-03	0.81			
		4	3.80E+05	2.86E+03	7.53E-03	0.90			
HEK293T	miRNA-αFoxg1.1694	1	3.07E+03	3.03E+05	1.01E-02	2.23	2.38	0.14	7.6E-05
		2	2.91E+03	2.65E+05	1.10E-02	2.42			
		3	1.94E+03	2.01E+05	9.65E-03	2.12			
		4	2.48E+03	1.98E+05	1.25E-02	2.75			
	miRNA.NC	1	7.90E+02	1.90E+05	4.16E-03	0.91	1.00	0.09	
		2	1.52E+03	2.89E+05	5.25E-03	1.15			
		3	7.61E+02	2.09E+05	3.64E-03	0.80			
		4	8.73E+02	1.69E+05	5.16E-03	1.13			
HEK293T	miRNA-αFoxg1.0650	1	2.24E+03	4.10E+05	5.48E-03	1.32	1.44	0.06	8.0E-03
		2	1.32E+03	2.11E+05	6.24E-03	1.50			
		3	1.46E+03	2.35E+05	6.22E-03	1.49			
	miRNA.NC	1	4.65E+02	1.35E+05	3.45E-03	0.83	1.00	0.09	
		2	1.63E+03	3.27E+05	4.98E-03	1.20			
		3	7.49E+02	2.11E+05	3.54E-03	0.85			
		4	1.30E+03	2.76E+05	4.69E-03	1.13			

PANEL S1C DATA

**RNAa in cell lines. Foxg1-protein levels**			protein (wb densitometry arbitrary units)		Foxg1 / Gapdh protein ratio	miR-NC-norm Foxg1 / Gapdh protein ratio			p-value (t-test)
cell line	sample		Foxg1	Gapdh		value	aver-age	sem	
NIH/3T3	miRNA-αFoxg1.1694	1	6.59E+01	1.22E+02	5.42E-01	2.04	1.98	0.27	4.3E-02
		2	9.33E+01	1.34E+02	6.97E-01	2.62			
		3	5.24E+01	1.01E+02	5.17E-01	1.94			
		4	4.69E+01	1.34E+02	3.51E-01	1.32			
	miRNA.NC	1	9.19E+00	1.42E+02	6.47E-02	0.24	1.00	0.40	
		2	1.81E+01	1.41E+02	1.29E-01	0.48			
		3	4.39E+01	1.28E+02	3.44E-01	1.30			
		4	5.07E+01	9.64E+01	5.26E-01	1.98			
HEK293T	miRNA-αFoxg1.1694	1	7.12E+01	8.91E+01	7.99E-01	3.08	4.30	0.72	4.0E-03
		2	6.91E+01	8.42E+01	8.21E-01	3.17			
		3	1.26E+02	9.86E+01	1.27E+00	4.92			
		4	1.58E+02	1.01E+02	1.57E+00	6.04			
	miRNA.NC	1	5.38E+01	9.07E+01	5.93E-01	2.29	1.00	0.45	
		2	2.45E+01	1.03E+02	2.37E-01	0.91			
		3	1.01E+01	1.03E+02	9.78E-02	0.38			
		4	1.05E+01	9.68E+01	1.09E-01	0.42			
HEK293T	miRNA-αFoxg1.0650	1	8.00E+01	1.04E+02	7.67E-01	2.01	1.59	0.17	1.5E-02
		2	5.69E+01	8.57E+01	6.64E-01	1.74			
		3	5.07E+01	9.77E+01	5.18E-01	1.36			
		4	5.12E+01	1.06E+02	4.85E-01	1.27			
	miRNA.NC	1	4.76E+01	1.43E+02	3.34E-01	0.87	1.00	0.12	
		2	5.05E+01	1.79E+02	2.82E-01	0.74			
		3	5.93E+01	1.36E+02	4.36E-01	1.14			
		4	6.07E+01	1.27E+02	4.76E-01	1.25			

PANEL S2 DATA

RNAa in (E12.5+DIV4) ncx precursors		number of amplicons		*AK158887*-ncRNA / *Gapdh*-mRNA amplicon ratio	miRNA-NC-norm *AK158887*-ncRNA / *Gapdh*-mRNA amplicon ratio			p-value (t-test, against miRNA-NC)
sample		*AK158887*-ncRNA	*Gapdh*-mRNA		value	aver-age	sem	
miRNA-αFoxg1.0650	a	3.94E+04	1.66E+07	2.38E-03	2.27	1.94	0.15	7.7E-08
	b	1.96E+04	1.09E+07	1.80E-03	1.72			
	c	9.37E+03	5.95E+06	1.58E-03	1.51			
	d	3.74E+04	1.74E+07	2.15E-03	2.06			
	e	1.63E+04	1.09E+07	1.50E-03	1.43			
	f	1.56E+04	1.00E+07	1.55E-03	1.48			
	g	1.77E+04	1.76E+07	1.00E-03	0.96			
	h	2.81E+04	1.27E+07	2.21E-03	2.11			
	i	4.37E+04	1.99E+07	2.20E-03	2.10			
	j	1.88E+04	6.19E+06	3.03E-03	2.90			
	k	3.99E+04	1.33E+07	3.00E-03	2.87			
	l	1.38E+04	7.06E+06	1.96E-03	1.87			
	m	3.06E+04	1.48E+07	2.07E-03	1.98			
miRNA-αFoxg1.1653	a	6.39E+03	8.67E+06	7.37E-04	0.70	0.93	0.12	3.3E-01
	b	7.28E+03	6.18E+06	1.18E-03	1.12			
	c	2.67E+03	2.70E+06	9.90E-04	0.95			
miRNA-NC	a	1.07E+04	1.24E+07	8.66E-04	0.83	1.00	0.06	
	b	5.60E+04	3.67E+07	1.53E-03	1.46			
	c	6.53E+03	8.74E+06	7.48E-04	0.71			
	d	1.82E+04	1.65E+07	1.11E-03	1.06			
	e	7.07E+03	5.98E+06	1.18E-03	1.13			
	f	1.42E+04	1.67E+07	8.51E-04	0.81			
	g	1.63E+04	1.59E+07	1.03E-03	0.98			
	h	3.16E+04	2.41E+07	1.31E-03	1.25			
	i	8.06E+03	1.00E+07	8.04E-04	0.77			
	j	2.49E+04	3.92E+07	6.36E-04	0.61			
	k	1.09E+04	1.03E+07	1.06E-03	1.01			
	l	1.87E+04	1.30E+07	1.44E-03	1.38			
	m	4.05E+04	2.59E+07	1.56E-03	1.49			
	n	4.93E+03	9.48E+06	5.20E-04	0.50			
	o	4.85E+03	4.58E+06	1.06E-03	1.01			
	p	4.84E+04	4.27E+07	1.13E-03	1.08			
	q	1.05E+04	1.50E+07	7.00E-04	0.67			
	r	1.24E+04	9.47E+06	1.31E-03	1.25			
	s	2.38E+04	2.00E+07	1.19E-03	1.14			
	t	1.26E+04	1.33E+07	9.48E-04	0.91			
	u	2.85E+04	2.84E+07	1.00E-03	0.96			

PANEL S3 DATA

basal RNApolII enrichment at the *Foxg1* locus (as evaluated in miR-NC-treated samples)			αRNApolII-IP / input amplicon ratio		
amplicon	exp	sample	value	aver-age	sem
(1)	Fig. 4F	a	0.027	0.018	0.003
		b	0.033		
		c	0.018		
	Fig. 4G	a	0.017		
		b	0.007		
		c	0.014		
		d	0.011		
(2)	Fig. 4F	a	0.011	0.010	0.001
		b	0.014		
		c	0.011		
	Fig. 4G	a	0.004		
		b	0.010		
		c	0.010		
(4)	Fig. 4F	a	0.021	0.017	0.003
		b	0.028		
		c	0.015		
	Fig. 4G	a	0.016		
		b	0.008		
		c	0.011		
(5)	Fig. 4F	a	0.018	0.016	0.005
		b	0.036		
		c	0.016		
	Fig. 4G	a	0.007		
		b	0.009		
		c	0.007		
(6)	Fig. 4F	a	0.062	0.038	0.008
		b	0.067		
		c	0.036		
	Fig. 4G	a	0.042		
		b	0.011		
		c	0.026		
		d	0.022		
(7)	Fig. 4F	a	0.045	0.034	0.008
		b	0.067		
		c	0.020		
	Fig. 4G	a	0.014		
		b	0.027		
		c	0.029		
(8)	Fig. 4F	a	0.042	0.026	0.006
		b	0.049		
		c	0.019		
	Fig. 4G	a	0.013		

		b	0.017		
		c	0.017		

PANEL S4B DATA

***Foxg1*-mRNA in transfected (E16.5+DIV3) ncx cultures**			number of amplicons		*Foxg1*-mRNA / *Gapdh*-mRNA amplicon ratio	ctr-norm *Foxg1*-mRNA / *Gapdh*-mRNA amplicon ratio			p-value
set	sample		*Foxg1*-mRNA	*Gapdh*-mRNA		value	aver-age	sem	
1	siRNA-αFoxg1.1694 (by lipo-fectamine)	a	1.0E+05	3.2E+05	3.2E-01	1.49	1.47	0.02	
		b	7.5E+04	2.3E+05	3.2E-01	1.48			
		c	3.9E+05	1.3E+06	3.1E-01	1.40			
		d	3.4E+05	1.0E+06	3.3E-01	1.50			
2	siRNA-αEGFP (by lipo-fectamine)	a	4.6E+04	2.1E+05	2.1E-01	0.98	1.13	0.06	*1-vs-2* 1.3E-03
		b	6.8E+04	2.6E+05	2.6E-01	1.20			
		c	3.4E+05	1.4E+06	2.4E-01	1.08			*1-vs-3* 5.9E-03
		d	2.6E+05	1.1E+06	2.3E-01	1.04			
		e	3.1E+05	1.1E+06	2.9E-01	1.33			
3	ctr (no lipo-fectamine)	a	4.5E+04	1.8E+05	2.5E-01	1.15	1.00	0.12	*2-vs-3* 1.9E-01
		b	6.2E+04	3.3E+05	1.9E-01	0.87			
		c	4.1E+05	1.4E+06	3.0E-01	1.39			
		d	2.9E+05	1.5E+06	2.0E-01	0.89			
		e	2.3E+05	1.5E+06	1.5E-01	0.70			

PANEL S4D DATA

***Foxg1*-mRNA in transfected (E16.5+DIV3) ncx cultures**			number of amplicons		*Foxg1*-mRNA / *Gapdh*-mRNA amplicon ratio	ctr-norm *Foxg1*-mRNA / *Gapdh*-mRNA amplicon ratio			p-value
set	sample		*Foxg1*-mRNA	*Gapdh*-mRNA		value	averag e	sem	
1	siRNA-αFoxg1.1694 (by RVG-R9)	a	8.3E+04	2.4E+05	3.4E-01	1.57	1.42	0.08	*1-vs-2* 5.0E-03 *1-vs-3* 9.0E-03 *2-vs-3* 2.2E-01
		b	1.8E+05	6.9E+05	2.6E-01	1.20			
		c	1.0E+05	3.1E+05	3.2E-01	1.47			
		d	7.2E+04	2.2E+05	3.2E-01	1.46			
2	siRNA-αEGFP (by RVG-R9)	a	7.9E+04	3.4E+05	2.3E-01	1.05	1.08	0.04	
		b	5.6E+04	2.3E+05	2.5E-01	1.11			
		c	7.9E+04	3.7E+05	2.1E-01	0.97			
		d	4.1E+04	1.6E+05	2.6E-01	1.19			
3	ctr (no RVG-R9)	a	4.5E+04	1.8E+05	2.5E-01	1.15	1.00	0.07	
		b	4.0E+04	2.2E+05	1.8E-01	0.83			
		c	2.2E+05	9.6E+05	2.2E-01	1.02			

PANEL S4F DATA

***Foxg1*-mRNA in transfected (E12.5+DIV7) ncx cultures**			number of amplicons		*Foxg1*-mRNA / *Gapdh*-mRNA amplicon ratio	ctr-norm *Foxg1*-mRNA / *Gapdh*-mRNA amplicon ratio			p-value
set	sample		*Foxg1*-mRNA	*Gapdh*-mRNA		value	aver-age	sem	
1	siRNA-αFoxg1.1694 (by RVG-R9)	a	5.6E+04	3.5E+05	1.6E-01	9.36	8.64	0.59	*1-vs-2* 3.1E-01
		b	3.8E+04	3.0E+05	1.3E-01	7.47			
		c	4.8E+04	3.1E+05	1.5E-01	9.09			
2	siRNA-αEGFP (by RVG-R9)	a	3.3E+04	2.5E+05	1.3E-01	7.78	9.15	0.73	*1-vs-3* 2.7E-05
		b	6.3E+04	3.6E+05	1.7E-01	10.28			
		c	4.7E+04	3.0E+05	1.6E-01	9.38			
3	ctr (no RVG-R9)	a	1.3E+02	9.9E+03	1.3E-02	0.77	1.00	0.29	*2-vs-3* 4.2E-05
		b	2.0E+03	1.6E+05	1.3E-02	0.75			
		c	1.0E+03	9.7E+04	1.0E-02	0.60			
		d	4.4E+03	1.4E+05	3.2E-02	1.87			
